# Metabolic QTL Analysis Links Chloroquine Resistance in *Plasmodium falciparum* to Impaired Hemoglobin Catabolism

**DOI:** 10.1371/journal.pgen.1004085

**Published:** 2014-01-02

**Authors:** Ian A. Lewis, Mark Wacker, Kellen L. Olszewski, Simon A. Cobbold, Katelynn S. Baska, Asako Tan, Michael T. Ferdig, Manuel Llinás

**Affiliations:** 1 Department of Molecular Biology and Lewis-Sigler Institute for Integrative Genomics, Princeton University, Princeton, New Jersey, United States of America; 2 Eck Institute for Global Health, Department of Biological Sciences, University of Notre Dame, Notre Dame, Indiana, United States of America; Broad Institute of Harvard and MIT, United States of America

## Abstract

Drug resistant strains of the malaria parasite, *Plasmodium falciparum*, have rendered chloroquine ineffective throughout much of the world. In parts of Africa and Asia, the coordinated shift from chloroquine to other drugs has resulted in the near disappearance of chloroquine-resistant (CQR) parasites from the population. Currently, there is no molecular explanation for this phenomenon. Herein, we employ metabolic quantitative trait locus mapping (mQTL) to analyze progeny from a genetic cross between chloroquine-susceptible (CQS) and CQR parasites. We identify a family of hemoglobin-derived peptides that are elevated in CQR parasites and show that peptide accumulation, drug resistance, and reduced parasite fitness are all linked *in vitro* to CQR alleles of the *P. falciparum* chloroquine resistance transporter (*pfcrt*). These findings suggest that CQR parasites are less fit because mutations in *pfcrt* interfere with hemoglobin digestion by the parasite. Moreover, our findings may provide a molecular explanation for the reemergence of CQS parasites in wild populations.

## Author Summary

Chloroquine was formerly a front line drug in the treatment of malaria. However, drug resistant strains of the malaria parasite have made this drug ineffective in many malaria endemic regions. Surprisingly, the discontinuation of chloroquine therapy has led to the reappearance of drug-sensitive parasites. In this study, we use metabolite quantitative trait locus analysis, parasite genetics, and peptidomics to demonstrate that chloroquine resistance is inherently linked to a defect in the parasite's ability to digest hemoglobin, which is an essential metabolic activity for malaria parasites. This metabolic impairment makes it harder for the drug-resistant parasites to reproduce than genetically-equivalent drug-sensitive parasites, and thus favors selection for drug-sensitive lines when parasites are in direct competition. Given these results, we attribute the re-emergence of chloroquine sensitive parasites in the wild to more efficient hemoglobin digestion.

## Introduction

Drug resistance is a critical issue facing worldwide malaria control. The spread and persistence of chloroquine-resistant (CQR) *Plasmodium falciparum* has rendered chloroquine, an inexpensive and potent drug, ineffective throughout most of the world [1]. In sub-Saharan Africa [2] and the island of Hainan (China) [3], where CQR parasites formerly accounted for 85–98% of the population, the coordinated cessation of chloroquine treatment resulted in a dramatic reduction (40–100%) in the prevalence of CQR parasites over 10 years. Although the reemergence of chloroquine sensitive (CQS) parasites is a major development with regard to human health, the underlying molecular mechanisms behind this phenomenon are unknown.

The importance of drug resistance to world health has prompted a half century of intensive research into the parasite's mechanisms of resistance [4]. These efforts identified the predominant gene responsible for chloroquine resistance, the *P. falciparum* chloroquine resistance transporter (*pfcrt, pf3D7_0709000*) [5]. PfCRT is a multiple pass membrane protein that is localized to the digestive vacuole [5], [6]. Mutations associated with chloroquine resistance have been mapped [7]–[10] and a single polymorphism in the first transmembrane domain (K76T) has been shown to be essential for drug resistance [11]. Recently, the resistant form of PfCRT was found to transport chloroquine under physiologically relevant conditions. Wildtype PfCRT is also assumed to function as a transporter [12], but its native substrates are unclear and the impact of CQR alleles on PfCRT's normal function remains a mystery.

Although mutations in *pfcrt* are necessary and sufficient to confer chloroquine resistance, several other genes have also been implicated in drug tolerance. The interactions between *pfcrt* and these other loci, including the *P. falciparum* multiple drug resistance gene (*pfmdr1*) [9], [13]–[15] and *P. falciparum* multiple resistance protein (*pfmrp1*) [16], are not clearly understood. One possibility is that mutations at secondary loci interact with *pfcrt* to modulate drug resistance. Alternatively, mutations at other loci may compensate for loss of function associated with CQR forms of PfCRT. Understanding how *pfcrt* mutations affect parasite physiology is an essential step towards unraveling these polygenic contributions to chloroquine resistance.

Given PfCRT's transmembrane structure, its localization to the digestive vacuole, and its ability to transport chloroquine *in vitro*, we hypothesized that wildtype PfCRT functions as a transporter. Furthermore, we predicted that mutations in *pfcrt*, and other CQR-associated genes, alter steady-state metabolite levels in PfCRT-associated pathways. Identifying these phenotypes and linking them to specific genes is difficult because 1) metabolites are often derived from multiple pathways, 2) steady-state levels of compounds can be affected by small perturbations far upstream or downstream of a particular compound and 3) metabolic regulation often involves complex interactions between nonlinear factors, such as covalent modification of enzymes, feedback inhibition, and allosteric regulation.

Quantitative trait locus (QTL) mapping is a powerful tool for unraveling complex metabolic networks and tracing metabolic regulation to specific genes [17], [18]. QTL mapping uses the segregation of alleles across a phenotypically and genetically diverse population to measure the genome-wide contribution of individual alleles to a phenotype (e.g. a metabolite concentration) [19]–[24]. Recently, this approach has been integrated with untargeted metabolomics to study metabolic regulation on a genome-wide scale [17]. This emerging metabolic QTL (mQTL) strategy is of obvious applicability to malariology. However, only three genetic crosses of *P. falciparum* have ever been completed because of serious logistical challenges [25]. One of these efforts crossed the CQS HB3 parasite clone with the CQR Dd2 clone. The haploid progeny from this cross provide a unique opportunity to investigate the metabolic consequences of drug resistance and the role of compensatory mutations in maintaining metabolic homeostasis.

In this study, we use high resolution mass spectrometry to measure the global metabolic profiles of progeny from the HB3×Dd2 genetic cross. Using mQTL mapping, we identify a family of hemoglobin-derived peptides that accumulate in parasites carrying CQR *pfcrt* alleles. We show that this phenotype can be recapitulated in transgenic parasite lines in which the native *pfcrt* gene has been replaced with a recombinant CQR or CQS *pfcrt* allele. In addition, we show that two independently evolved CQR alleles of *pfcrt* confer a fitness cost. From these data, we propose that CQR imparts a fitness cost on parasites by disrupting hemoglobin catabolism.

## Results

### Mapping metabolite levels to genetic loci

**Figure 1:**
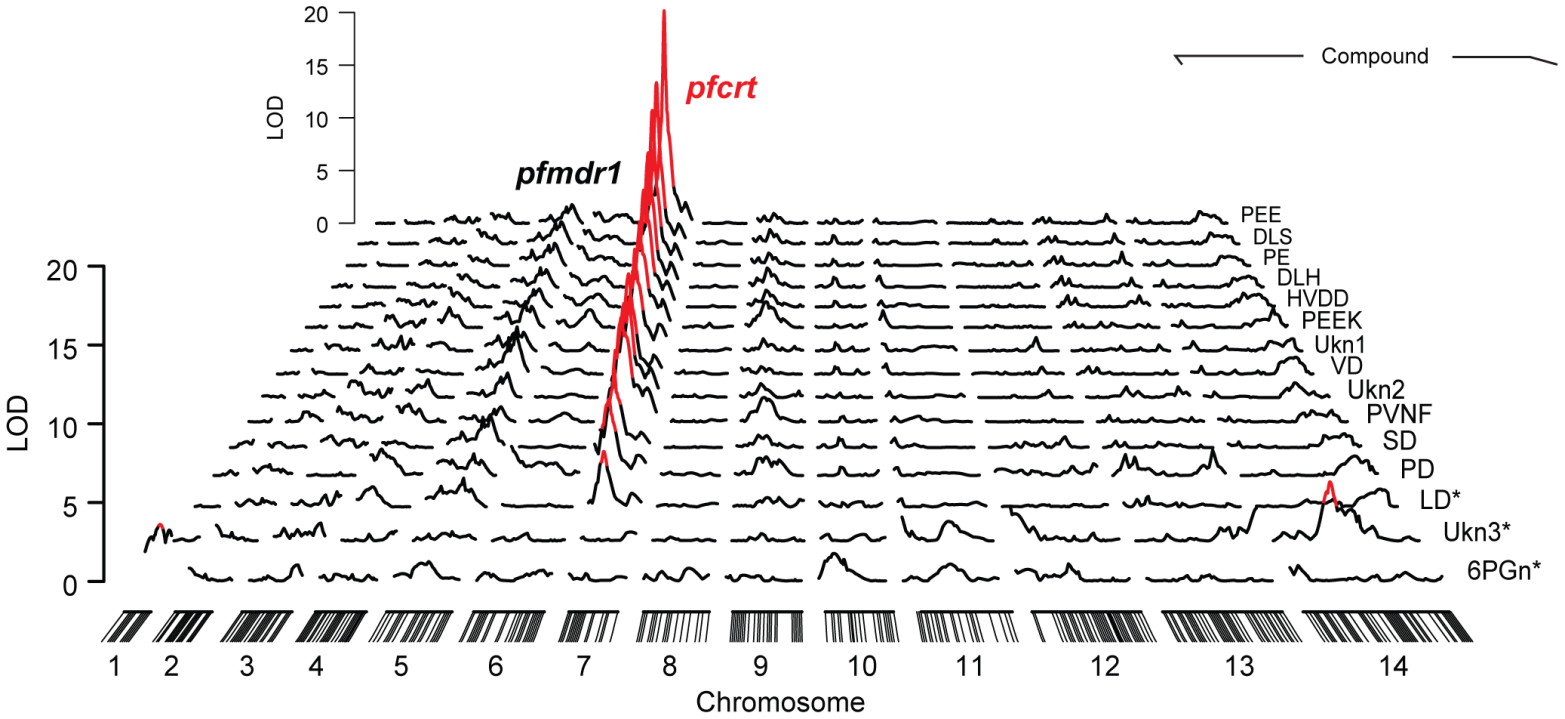
Significant genome-wide mQTL hits. Significance scores were calculated from the natural log of the median compound intensity observed in triplicate pool replicates. All markers showing significant associations (genome-wide α = .05, n = 35) are colored in red and hash marks on the x axis denote the positions of the microsatellite markers. The mean location of the chromosome 7 associated peaks is 22.0 cM, which contains *pfcrt* and eight other genes. The secondary LOD peak observed on chromosome 5 is consistent with *pfmdr1*, another CQR-related locus. Peptides are listed by their standard abbreviations, unknowns are abbreviated as Ukn, and 6PGn indicates 6-phosphogluconate. Compounds were identified by ms/ms fragmentation and coelution assays (Figure 2). *All compounds except LD, Ukn3, and 6PGn had significant peak LOD scores after Bonferroni correction for multiple hypothesis testing (α = 0.05/24).

We combined genome-wide QTL mapping with mass spectrometry (MS)-based metabolomics to identify genetic loci in *P. falciparum* that have a significant influence over steady-state metabolite levels. To achieve this, synchronous trophozoite-stage cultures (24 hour post invasion) of the 34 haploid progeny and two parental lines from the HB3×Dd2 genetic were grown using established *in vitro* methods [26]. Metabolites were harvested from each of the cultures and high-resolution mass data were collected on an LTQ-Orbitrap. For maximum sensitivity, mass data were peak-picked near the noise threshold (minimum signal/noise = 3) and biologically relevant data were identified using a two-stage assignment routine. In the first stage, promising signals in the untargeted mass list (N = 124,020) were identified by QTL analysis and genetic linkages with LOD scores greater than 3 (N = 1,707) were manually curated to remove artifacts and correct for errors in the automated MS data peak picking algorithm. Untargeted mass data are highly redundant because electrospray ionization generates numerous adducts and in-source fragments for each input metabolite. Consequently, we employed a second assignment stage to condense redundant data into a single representative parent mass for each compound. Curated signals (N = 279) were clustered by coelution, signal covariance, and mass difference relative to common adducts/isotopomers/fragments. The most intense signal from each group was designated as the parent mass. A total of 15 signals passed this two-stage filtering routine. Each of these signals had LOD scores above their permutation-established thresholds for genome-wide significance (α = .05), and all but 3 of these signals were significant after Bonferroni correction (α = .05/24) for multiple hypothesis testing (Table 1). Notably, all but one of these 15 signals have LOD scores above the 5% false discovery threshold (LOD = 5.5) established by Q-value analysis for the original unfiltered mass list. Surprisingly, all Bonferroni-compliant signals were linked to a single 22 cM genomic region on chromosome 7 (Figure 1) containing *pfcrt*, the gene responsible for conferring chloroquine resistance [5].

**Table 1:** Genetic linkage by QTL and concentrations of metabolites‡ (µM ± s.d.) observed in parasite extracts from chloroquine sensitive (CQS) and resistant (CQR) transgenic parasites.

Compound	Chr	Pos	LOD	α*0.05	α*.05/24	*p*-value(QTL)**	CQS †C2 µM	CQR †C4 µM	CQR †C6 µM	*p*-value**C2∶C4∶C6
PEE	7	20	20.2	3.6	6.1	<.001	0.5±0.09	5.1±0.4	5.4±0.3	5.4E-06
DLS	7	20	14.9	3.4	4.9	<.001	0.46±0.1	9.7±0.9	11.6±0.2	7.1E-07
PE	7	20	14.0	3.6	5.6	<.001	1.4±0.04	36.6±3.3	15.2±0.4	3.9E-06
DLH	7	22	11.7	3.4	4.7	<.001	0.35±0.03	16.8±1.5	8.4±0.3	4.5E-06
HVDD	7	21	9.9	3.0	5.0	<.001	0.29±0.09	5.1±0.6	4.3±0.2	1.7E-06
PEEK	7	23.1	8.7	3.3	5.2	<.001	0.11±0.05	0.59±0.08	0.65±0.08	3.2E-04
Ukn2	7	23.1	8.2	3.4	5.2	<.001	-	-	-	-
VD	7	22	7.9	3.2	4.8	<.001	0.23±0.02	1.1±0.1	0.74±0.03	9.5E-05
Ukn1	7	28.9	7.3	3.3	5.4	<.001	-	-	-	-
PVNF	7	22	7.1	3.3	4.7	<.001	0.07±0.02	1±0.2	0.79±0.14	5.8E-04
SD	7	23.1	5.5	3.1	4.8	<.001	0.85±0.17	2.8±0.5	2±0.3	0.004
PD	7	22	5.3	3.1	4.8	0.001	38.5±0.4	75.7±4.8	27.8±2.2	5.6E-06
LD	7	23.1	3.7	2.8	4.6	0.012	0.1±0.02	0.58±0.18	0.53±0.15	0.012
Ukn3	14	84	3.8	3.4	4.6	0.022	-	-	-	-
6PGn	1	21	3.5	3.3	4.5	0.034	1.4±0.3	1.8±0.4	1.3±0.1	0.045

Peptides are listed by their standard abbreviations, unknowns are abbreviated as Ukn, and 6PGn indicates 6-phosphogluconate.

Threshold LOD scores for genome wide significance, computed by permutation, before and after Bonferroni α correction for multiple comparison testing. Q-value analysis of the original unfiltered mass list established a LOD score of 5.5 as the 5% false discovery threshold.

*p*-values for QTL analysis reflect the genome-wide significance computed by permutation (N = 1000) of the sample genotypes (N = 36); *p*-values reported for metabolite concentrations observed in the parasite extracts were computed by one-way analysis of variance (N = 9; K = 3).

Metabolite levels are reported as µM concentrations present in 1∶4 final dilutions of extracts from packed infected cells (∼90% parasitemia) as measured by single reaction monitoring mass spectrometry. C2 parasites carry the CQS HB3 *pfcrt* allele, C4 and C6 carry the CQR *pfcrt* alleles from Dd2 and 7G8, respectively.

### Identifying PfCRT-linked metabolites

**Figure 2:**
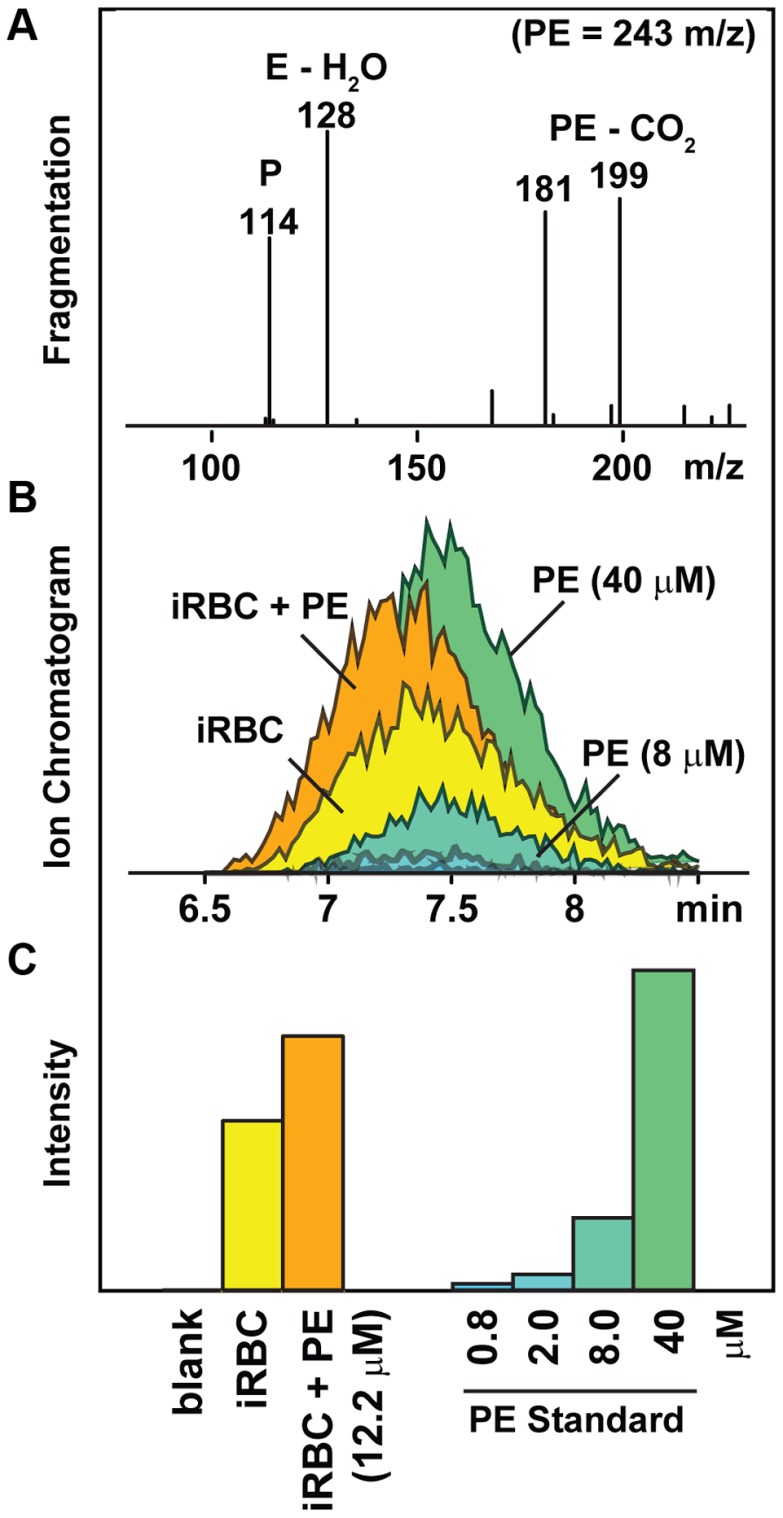
Strategy for identifying unknown compounds. A representative dataset used to identify the dipeptide prolylglutamate (PE) is shown. Putative compound identities were assigned by exact mass matching followed by (A) ms/ms fragmentation analysis of extracts from purified infected red blood cells (iRBC). These assignments were confirmed by (B) chromatographic coelution of the parasite-derived compound with a standard, and (C) a concentration-dependent change in the target compound's intensity when a standard was added to the iRBC extract. Mass fragments are listed by their negative-mode mass (m/z −H^+^). The 114 m/z fragment shown in panel A was used for identification and quantification of the PE peptide (Table 1).

Tentative metabolite assignments were generated for each of the PfCRT-linked masses by submitting observed signals to the Madison Metabolomics Consortium Database [27] and the Human Metabolome Database [28]. The resulting list of putative IDs was evaluated by analyzing ms/ms fragmentation spectra from enriched parasite extracts (Figure 2). Eleven of the 15 significant compounds (α = .05) had exact masses and fragmentation spectra consistent with small peptides. Peptide assignments were empirically validated by co-elution of the parasite-derived signal with synthetic peptide standards. These experiments were conducted using single reaction monitoring (SRM) mass spectrometry, a robust analytical method for confirming specific metabolite assignments in complex mixtures [29]. Each of the PfCRT-linked peptides co-eluted with their respective synthetic standards. Moreover, the intensity of the parasite-derived signal changed in a concentration dependent manner when standards were added to parasite extracts (Figure 2).

### Validating the PfCRT/peptide linkage

**Figure 3:**
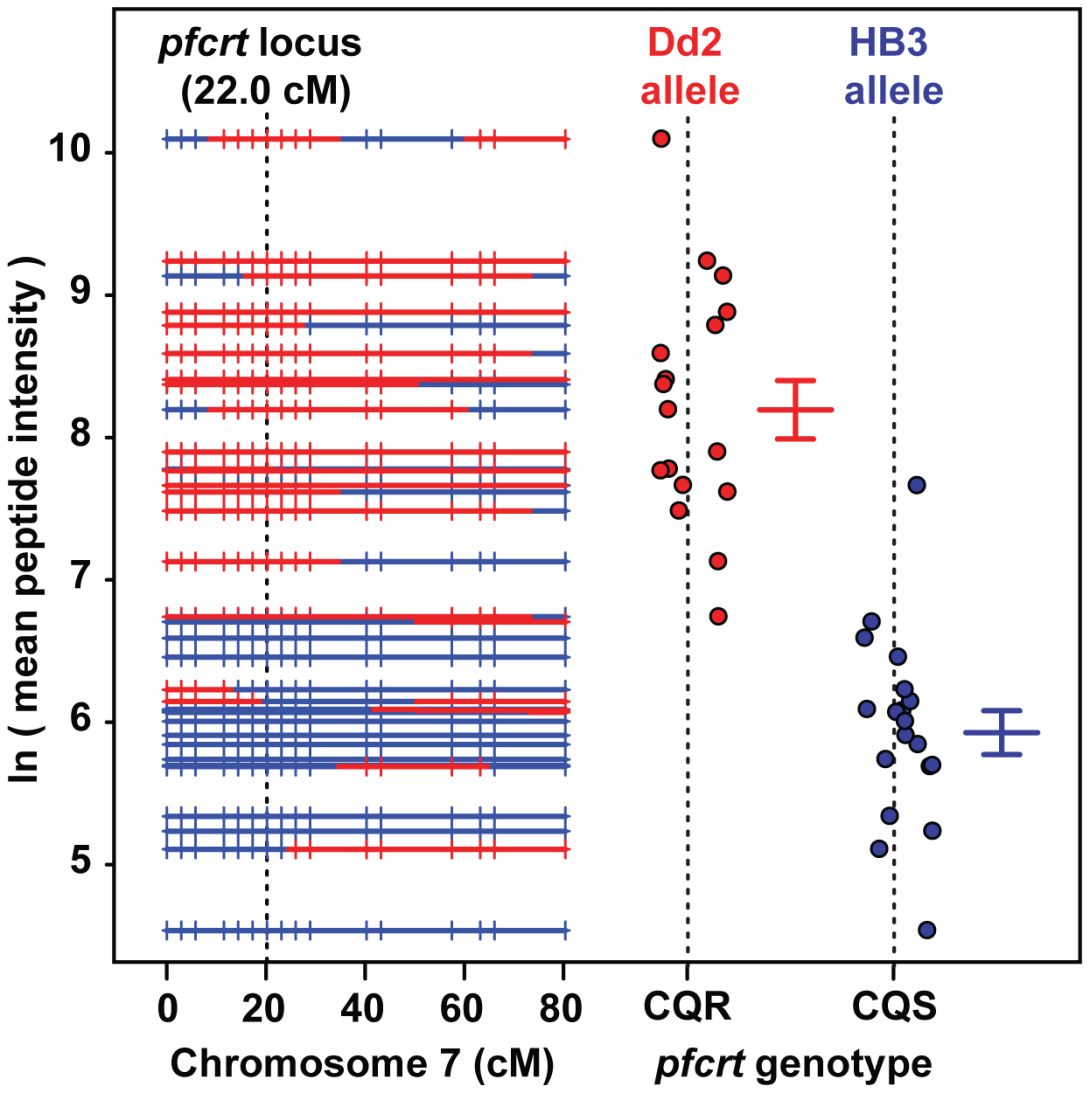
Peptide accumulation co-segregates with the *pfcrt* locus on Chromosome 7. A genetic recombination map is plotted versus the mean *pfcrt*-linked peptide level observed with each clone. Progeny carrying the Dd2 (red) chloroquine resistant allele at the *pfcrt* locus show higher levels of peptides than progeny carrying the HB3 chloroquine sensitive allele (blue). The mean peptide intensity used for the y-axis was computed from all of the covariate *pfcrt*-linked signals (Figure S1). The hash marks shown on each of the chromosomes indicate the positions of microsatellite markers used in this analysis.

**Figure 4:**
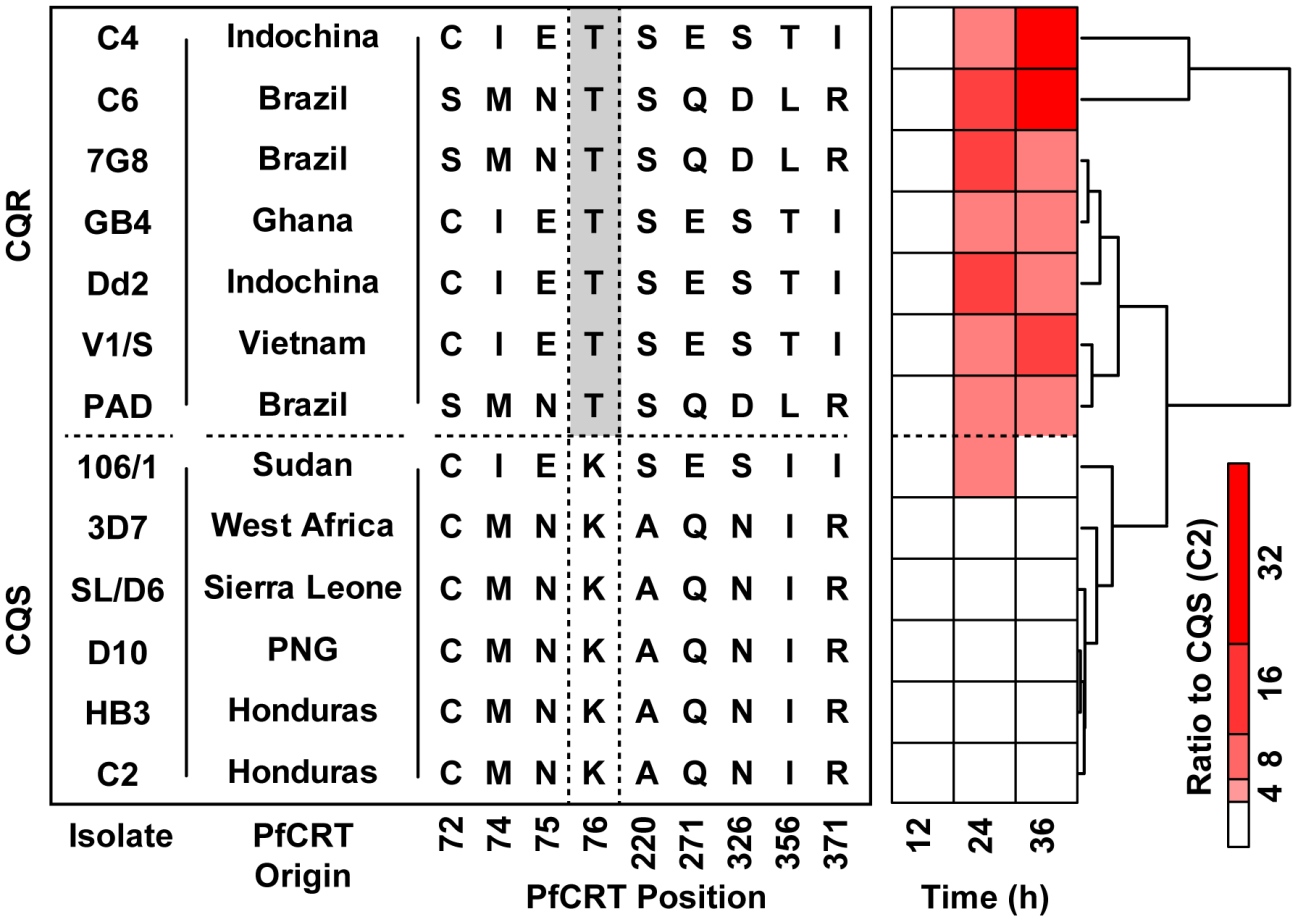
Geographically diverse isolates carrying the PfCRT-K76T polymorphism accumulate peptides. The K76T mutation is necessary to confer chloroquine resistance. The isolate nomenclature, geographical origin of the *pfcrt* allele, genotype at key residues, and the mean peptide signal observed in each isolate are shown. The mean peptide signal is a composite phenotype reporting the average intensity of 12 *pfcrt*-linked signals identified by mQTL (see Figure S1 for individual peptide profiles). All signals are expressed as a fold change relative to the maximum corresponding intensity observed in the CQS C2 line. The polymorphisms and origins of these lines were adapted from a previous publication [30].

QTL analysis demonstrated that the peptide accumulation phenotype observed in CQR parasites maps to a 36 kb locus on chromosome 7 containing *pfcrt* and eight other genes (Figure 3). To determine if the *pfcrt* gene is responsible for the peptide accumulation phenotype, we analyzed transgenic parasites in which the native *pfcrt* gene has been replaced with either a CQS (C2; HB3 allele) or CQR (C4, Dd2 allele; C6, 7G8 allele) variant of *pfcrt* [11]. The two CQR alleles we tested have distinct evolutionary origins but all of the transgenic parasites share the CQS GC03 background (a progeny clone from the HB3×Dd2 cross). MS analysis demonstrated that parasites carrying either of the CQR *pfcrt* alleles accumulate peptides over the 48-hour intraerythrocytic life cycle to much higher levels (>32-fold) than parasites carrying the CQS allele (Figure S1). Furthermore, a survey of diverse parasite genotypes showed that all of the parasites that accumulate peptides carry the critical PfCRT-K76T polymorphism that is required for chloroquine resistance [30] (Figure 4).

### Peptidomics analysis of parasites

**Figure 5:**
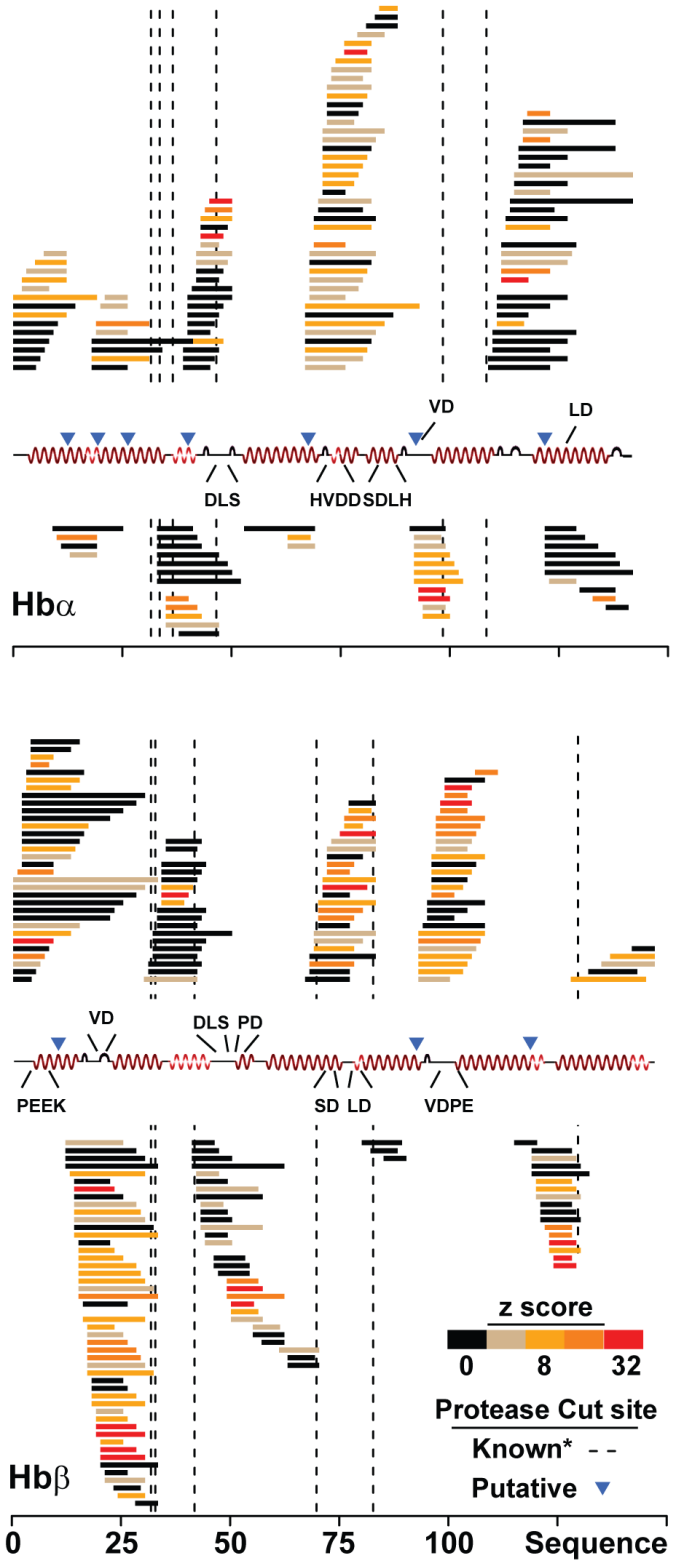
CQR isoforms of PfCRT disrupt hemoglobin metabolism. Endogenous peptides present in CQS (C2) versus CQR (C4 or C6) PfCRT allele-exchange parasites lines were identified by LC-MS/MS peptidomics and mapped to their corresponding locations on the α and β hemoglobin primary sequence. Peptides are colored by their mean absolute z-score: mean(|(C4_pep_-C2_pep_)/σ_pep_|, |(C6_pep_-C2_pep_)/σ_pep_|). The *pfcrt*-linked sequences that were identified by mQTL are denoted above the hemoglobin secondary structure. *Known protease cut sites depicted here were taken from published sites [33]. The sequence of each peptide is available in Figure S3, Figure S4, and Table S1.

Hemoglobin catabolism is an essential activity that provides amino acids and the physical space parasites need to grow [31]. All of the PfCRT-linked peptides identified by QTL analysis are found in, but not unique to, hemoglobin. Because PfCRT is located on the digestive vacuole membrane, which is the organelle where hemoglobin metabolism occurs, we hypothesized that PfCRT polymorphisms directly affect hemoglobin catabolism. To test this hypothesis, we conducted a comprehensive peptidomics analysis of parasites to monitor PfCRT-related effects on the hemoglobin digestion pathway. Erythrocytes infected with either CQS (C2) or CQR (C4, C6) parasites were purified by Percoll density gradient and endogenous peptides present in parasite extracts were analyzed by high-resolution nanospray LC-MS/MS. This analysis identified 362 endogenous peptides ranging from trimers to 32-mers that exactly correspond to sequences found in either the α or β chain of hemoglobin (Figure 5, Figure S3, and Figure S4). The majority of these peptides (e.g. VHLTPEE) have sequences that are unique to hemoglobin (i.e. have no other exact matches in either the *P. falciparum* or human genomes) and exist in overlapping clusters of structurally related peptides. The peptide clustering we observed is consistent with the parasite's semi-ordered hemoglobin digestion cascade, which involves protein degradation via a series of proteases (plasmepsins, falcipains, and falcilysin) and aminopeptidases [32]. The boundaries of most of the peptide clusters we observed coincide with established proteolytic cleavage sites [33]. In addition, we observed several sequence breaks that may suggest previously unmapped cut sites (Figure 5). Quantitative analysis identified 87 peptides that show evidence for differential accumulation between CQS and CQR lines (|z-score|>4; Figure 5). These peptides include the mQTL-linked peptides (e.g. PEE and DLS), other structurally related peptides (e.g. VHLTPEEK and HFDLS), and novel classes of peptides that fell below the analytical limit of detection in the original mQTL analysis (e.g. DPENFR in β-hemoglobin).

### Metabolomics analysis of transgenic parasites

We interpret peptide accumulation in CQR parasites as evidence for impaired hemoglobin catabolism. Because hemoglobin catabolism is essential to *P. falciparum* [31], we anticipated that CQR parasites may have alterations in other metabolic pathways. To determine the degree to which CRQ *pfcrt* alleles affect metabolic homeostasis, we quantified metabolites present in extracts from density-purified samples of RBCs infected with transgenic CQS (C2) and CQR (C4, C6) allele exchange parasites. Using high resolution HPLC-MS, we quantified 80 metabolites, including representative central carbon metabolites, nucleotides, cofactors, amino acids, and peptides. Surprisingly, the only compounds showing consistent steady-state metabolic differences between the CQS and CQR lines were hemoglobin-derived peptides (Figure S5). These data suggest that altered hemoglobin catabolism is the most significant metabolic consequence of CQR mutations.

### Fitness cost of chloroquine resistance

**Figure 6:**
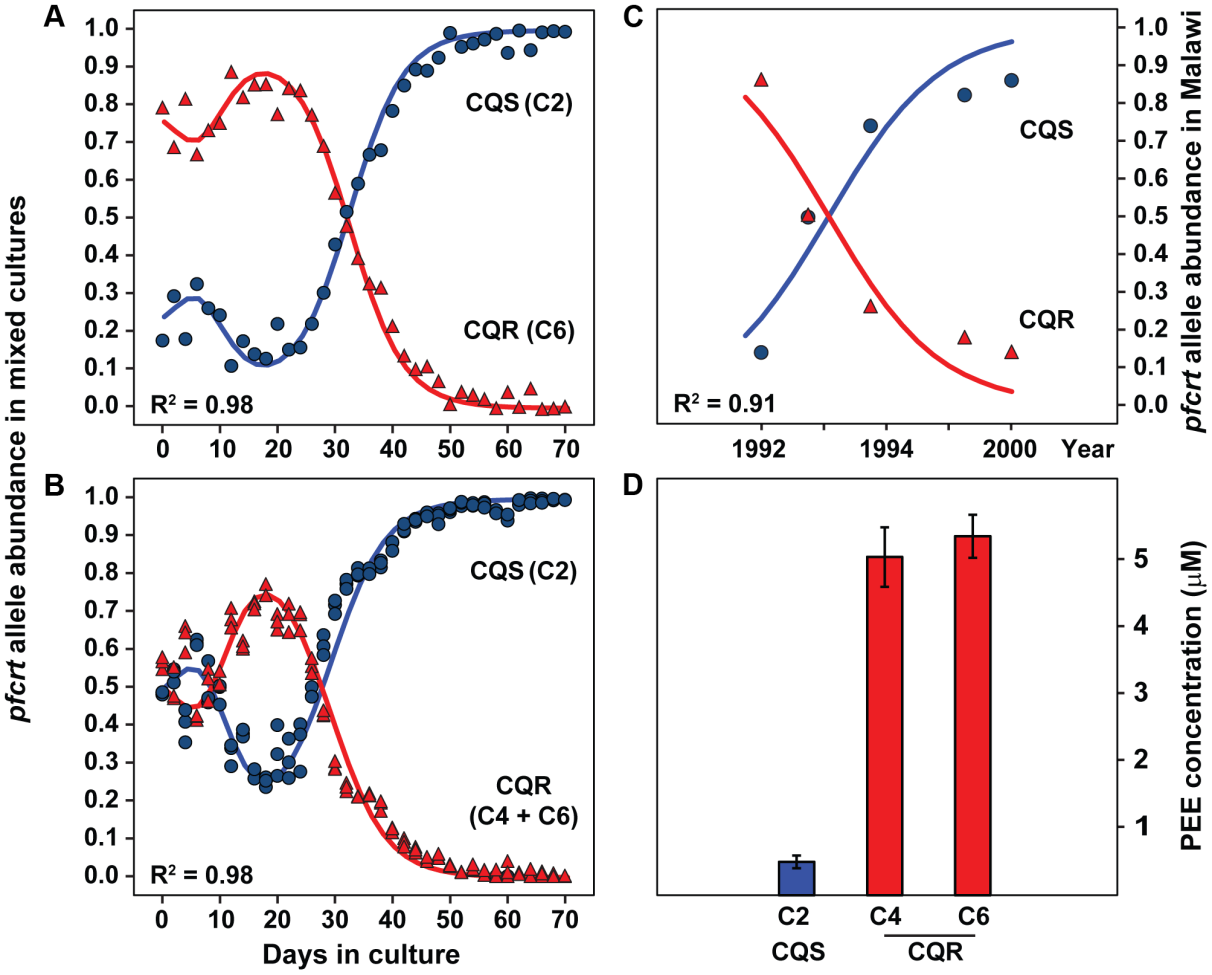
Chloroquine sensitive alleles outcompete resistant alleles *in vitro* and in endemic populations. Transgenic parasites carrying either a CQS (C2, HB3) or CQR (Dd2, C4; 7G8, C6) allele of *pfcrt* were mixed in culture flasks and competed head-to-head for 70 days (∼35 generations) *in vitro*. (**A**) Culture flasks were prepared with either 2 (C2, C6) or (**B**) 3 (C2, C4, C6) transgenic parasite lines and were maintained using standard culturing conditions. DNA was harvested every 48 h and the allelic composition of each flask was determined by quantitative DNA sequencing. The peak CQR DNA abundance at 18 days and the subsequent disappearance of CQR alleles are consistent with an *in vitro* competition model that allows for differences in lifecycle length and overall fitness (R^2^ = .98, Δ2 h cycle length and 13% differential fitness). Validation of the quantitative method and a comprehensive panel of the 2-way competition data are provided in Figure S7 and Figure S8. (**C**) Reemergence of CQS parasites in a wild population of parasites in Malawi following the cessation of chloroquine treatment. The Malawi data were adapted from Kublin et al. [2] and are reproduced with permission from Oxford University Press. The regression line shows the best fit reciprocally-adjusted exponential competition curve. (**D**) Concentrations of the tripeptide proline-glutamate-glutamate (PEE) in metabolite extracts from red blood cells infected with transgenic parasites carrying either CQS or CQR alleles of *pfcrt*.

Given the significance of hemoglobin catabolism in the parasite's blood stage development, we hypothesized that CQR-induced peptide-accumulation would be associated with a fitness cost. To test this hypothesis, we conducted long-term competition experiments between CQS and CQR transgenic parasites grown for 70 days in mixed cultures containing either two (C2, C4; C2, C6) or three (C2, C4, C6) allele-exchange parasite lines in the same flask. This experiment differs from previous *in vitro* studies in our use of the transgenic *pfcrt* allele exchange parasites, which controls for the polygenic contributions from genetic background, and the long timeframe over which cultures were allowed to compete (∼35 generations). DNA was harvested every 48 hours and the abundance of each *pfcrt* allele was quantified by Sanger sequencing (Figure S6 and Figure S7). Quantitative DNA sequencing showed that mixed populations of CQS/CQR parasites converted to nearly pure populations of CQS parasites after 70 days (Figure 6). This result was consistent across both Asian (Dd2, C4) and South American (7G8, C6) CQR *pfcrt* alleles and the outcome was not influenced by the starting ratio of the mixed populations (Figure 6 and Figure S8).

Our competition assay showed a transient increase in CQR allele abundance that peaked at ∼20 days. This transient peak is attributable to a difference in cell cycle length between CQR and CQS parasites. Differences in cycle length accumulate across generations and thus progressively offset cycle stages of competing populations. Since parasites amplify their DNA midway through their 48 hour cell cycle, and daughter progeny do not all successfully re-invade, the population with the longer generation-to-generation replication time will have more DNA (but fewer infected cells) when populations are offset across generations (i.e. the leading population is at the ring stage whereas the trailing population is at the schizont stage). To quantify this phenomenon, we constructed a computer model of *in vitro P. falciparum* competition. Our model showed that a 2 hour difference in cell cycle length and a 13% overall fitness cost per generation can explain both the transient increase in CQR DNA and the subsequent disappearance of CQR alleles from mixed cultures (R^2^ = .98, Figure 6). This computational assessment is empirically supported by competition experiments involving asynchronous populations of parasites, which showed the same long-term population dynamics but lack the transient increase in CQR allele abundance (Figure S8).

Since CQR parasites are less fit (Figure 6 and Figure S8) and have altered hemoglobin metabolism when compared to CQS parasites (Figure 6), we predicted that CQR parasites would have more difficulty in using hemoglobin-derived amino acids for biomass production. To test this, growth assays were conducted in a modified RPMI medium lacking all amino acids except isoleucine (which is not present in hemoglobin), which forces parasites to use hemoglobin to supply its amino acid needs. In agreement with the literature [31], [34] parasites incubated in isoleucine-only medium grew more slowly than those incubated in rich medium. However, CQR parasites were significantly more impaired than CQS parasites (*p* = 0.0075, Figure S9), suggesting that CQR-induced fitness changes are linked to hemoglobin catabolism.

## Discussion

This study provides four independent lines of evidence linking chloroquine resistance to hemoglobin catabolism: I) mQTL analysis demonstrated that elevated hemoglobin-derived peptides co-segregate with the CQR-encoding *pfcrt* locus (Figure 1 and Figure 3), II) levels of hemoglobin-derived peptides observed in parasite extracts predict chloroquine susceptibility in genetically diverse parasite strains from around the world (Figure 4), III) genetically identical parasite lines that differ only by CQR versus CQS isoforms of PfCRT recapitulate the peptide phenotypes observed in wild isolates (Figure S1 and Figure S2), and IV) forcing parasites to rely on hemoglobin as an amino acid source affects CQR parasites more severely than CQS parasites. In addition, we show that chloroquine resistant alleles affect the levels of 87 of the 362 observable peptides in the parasite's hemoglobin catabolism pathway (Figure 5). Finally, we demonstrate that the peptide accumulation phenotype is associated with a 2 hour increase in cell cycle duration and a 13% overall fitness cost in transgenic parasites that only differ by their *pfcrt* allele. Together, these data argue that the significant fitness disadvantage observed in CQR parasites is attributable to impaired hemoglobin metabolism.

These results provide the first molecular explanation for the reemergence of CQS parasites in wild populations following the cessation of chloroquine treatment. Parasites degrade ∼75% of the host cell hemoglobin over the course of their 48 hour intraerythrocytic developmental cycle [35], [36]. Any impairment in the hemoglobin digestion pathway directly affects I) the amino acid pool available for new protein synthesis [32], II) the osmotic stability of infected cells [37], and III) may reduce the number of developing merozoites that can fit within the physical confines of the infected erythrocyte. Any of these mechanisms could account for the increased cycle length and lower generation-to-generation fecundity we observed [37]. Although this is the first report of a metabolic perturbation inherent to CQR alleles of *pfcrt*, similar fitness-linked phenomena are associated with other drug resistance genes [13], [38]. Epidemiological analysis of the parasite population changes in Malawi and Hainan estimated the fitness cost of CQR to be ∼5% [39], [40]. Our *in vitro* competition studies support this conclusion and show that the fitness cost may actually be much higher in the absence of compensatory mutations (Figure 6).

While PfCRT isoforms are clearly the main contributor to the peptide-accumulation phenotype, our data also show that genetic background modulates PfCRT's effects on hemoglobin metabolism. The wide distribution of peptide levels observed across the HB3×Dd2 progeny (Figure 3), and the consistent secondary peaks observed in the mQTL analysis (Figure 1), suggest that loci other than PfCRT are contributing to peptide accumulation. Similarly, the CQR allele-exchange parasite lines (C4 and C6) both showed more extreme phenotypes than their respective parental lines (Dd2 and 7G8; Figure 4) despite having identical *pfcrt* sequences. The PfCRT/hemoglobin catabolism link we describe here, along with our peptidomics approach, provide a framework for investigating compensatory mutations elsewhere in the genome.

Identifying the native function of PfCRT is a subject of considerable interest to the parasitology community. One possible interpretation of the peptide accumulation phenotype is that wildtype PfCRT functions as a peptide transporter and that CQR mutations interfere with this activity [41]. This interpretation is supported by a recent report of glutathione transport in *Xenopus* oocytes expressing PfCRT [42], and by work in *Arabidopsis*, which showed that a plant PfCRT homolog mediates glutathione transport [43]. However, the broad diversity of sizes (2–32mers) and physical properties of peptides accumulated by CQR parasites are inconsistent with the relatively narrow range of substrates carried by most peptide transporters [44]. An alternative interpretation of the peptide phenotype is that CQR-associated mutations affect hemoglobin catabolism indirectly by altering the permeability of the digestive vacuole membrane. Resistance mutations in PfCRT, and perhaps other membrane proteins, may cause the digestive vacuole to leak protons [45], glutathione [42], heme, or other osmolytes, which thereby alter the solution conditions of the vacuolar compartment. Given that protease activity can be very sensitive to solution conditions [46], even modest changes in vacuolar conditions could interfere with hemoglobin catabolism by the parasite. Similarly, perturbations in solution conditions may affect protein-protein interaction and thereby disrupt the recently described hemoglobin degradation complex [47].

In conclusion, this study demonstrates that chloroquine resistance, impaired hemoglobin catabolism, and reduced parasite fitness are linked to polymorphisms in PfCRT. This surprising linkage provides a molecular explanation for the reemergence of CQS parasites in Africa and Asia. Our results suggest that co-formulating chloroquine with a *P. falciparum* protease inhibitor [48] may be an effective strategy for slowing the emergence of resistant parasites.

## Materials and Methods

### Parasite culture

Synchronous parasites were grown using established methods [26] in RPMI 1640 supplemented with 25 mM HEPES, 100 µM hypoxanthine (all from Sigma), 10 µg/mL gentamycin (Gibco) and 2.5 g/L Albumax II (Gibco). A total of 47 parasite strains were analyzed in this study; these strains include 36 lines from the HB3×Dd2 cross (34 progeny and 2 parental), three *pfcrt* allele swap lines (C2, C4, and C6) prepared in an isogenic GC03 background [11], and 8 out-group lines (V1/S, PAD, 7G8, GB4, 3D7, D10, SL/D6, and 106/1) used to measure metabolic phenotypes in diverse genetic backgrounds. All cultures were maintained at 5% hematocrit in a 37°C incubator with an atmosphere of 5% CO_2_, 6% O_2_, and 89% N_2_. Cultures were triple synchronized using consecutive treatments with 5% sorbitol at 0, 48, and 56 h of culture. Invasion time was determined by preparing blood smears every two hours starting 34 h after the last sorbitol treatment. The zero time point was designated when cultures reached 95% rings. Samples were harvested at 24 h post invasion for the HB3×Dd2 cross study, 38 h for the parasite enrichment studies, and at several time points throughout the cyclic 48-hour asexual blood stage (12, 24, and 36 h) for the out-group analysis.

### Purifying infected cells

To confirm our PfCRT-related phenotypes and improve our analytical sensitivity, erythrocytes infected with late trophozoite-stage parasites (38 h post invasion) were separated from uninfected erythrocytes using an established density gradient method [49]. Briefly, bulk cultures were suspended at 30% hematocrit in RPMI. Cultures were layered over dual Percoll layers (70% lower, 30% upper) diluted in 1× RPMI (final concentration). Samples were centrifuged (2,000× g, 15 min) and the infected erythrocyte layer was collected from the 30%/70% interface. Infected cells were washed with 50 volumes of RPMI, then suspended at 0.4% hematocrit in RPMI. Parasitemias of the purified samples were checked by blood smear and the purified samples were allowed to recover for 4 h in a 37°C incubator prior to metabolite extraction (Text S1 provides a step-by-step protocol).

### Metabolite extraction

Our metabolite extraction protocol is adapted from a previously established method [50]. Metabolites were extracted by suspending 50 µL packed cells in 1 mL 4°C 90% methanol. Samples were vortexed and briefly sonicated, if necessary, to disrupt the cell pellet and generate a uniform homogenate. Homogenates were centrifuged (13,000× g, 10 min) and the supernatants were harvested. Samples were stored at −80°C as 90% methanolic extracts until metabolite analysis. Just prior to analysis, extracts were dried under a stream of N_2_ gas and resuspended in 200 µL H_2_O (Text S1 provides a step-by-step protocol).

### Mass spectrometry

Metabolite extracts were analyzed by high performance liquid chromatography (HPLC) mass spectrometry (MS). The chromatographic conditions used in this study have been described in detail elsewhere [50]. Briefly, metabolites were separated by reverse phase C18 chromatography run over a 50 minute (HB3×Dd2 cross study and coelution assays) or 25 minute gradient (all other studies) using tributylamine as an ion pairing agent. General metabolite analyses were conducted using negative-mode electrospray ionization on a Thermo Scientific LTQ-Orbitrap (HB3×Dd2 cross) or Thermo Exactive (all other studies). Metabolite assignments were validated by single reaction monitoring (SRM) on a Thermo TSQ Quantum Discovery Max triple quad. For peptidomics analysis, aliquots of each metabolite extract were harvested, diluted 1∶4 in a 3% acetonitrile and 0.1% formic acid solution (final concentrations), and analyzed in positive mode on an LTQ-Orbitrap using nanospray from a 120 minute hydrophilic interaction liquid chromatography (HILIC) gradient. Scans were conducted at both low (150–500 m/z) and high (450–1800 m/z) mass ranges to accommodate multiple charge states and MS2 scans were automatically conducted on fragments from each of the top seven signals observed at any given time. Both the original cross dataset and the peptidomic analyses were collected at the Princeton mass spectrometry facility; all other data were collected in house.

### QTL analysis

Genome-wide scans were performed using pseudomarker [51] to detect QTLs associated with metabolite levels in the HB3×Dd2 genetic cross. Intensities of mass signals were log-transformed and the median signal for each mass across replicates was used as a phenotype. Batch number was included as an independent covariate [52] to correct for run-to-run changes in MS instrument sensitivity. Genome-wide significance thresholds were determined by permutation testing (*n* = 1000 permutations) [53] and the strength of each linkage was expressed as a LOD score. We accounted for multiple hypothesis testing using established methods [21]. Briefly, false discovery rates were calculated from *p*-values using the *q*-value approach [54]. QTL-based significance scores were used to filter the large untargeted mass list to a manageable subset of putative signals. Final LOD scores and significance thresholds were computed using R/qtl [55] (interval mapping parameters step = 1, n.draws = 64; QTL mapping method = hk). The custom R code (Text S2 and Text S3) and data tables used for this analysis (Table S2 and Table S3) are included in the supplemental materials.

### Metabolomics data analysis

Mass data were visualized and analyzed using MAVEN, a freely available software package for MS-based metabolomics [56]. Data were peak picked using a permissive threshold (S/N = 3) and raw LOD scores generated by QTL mapping were then used to identify the most promising subset of signals. The extracted ion chromatogram of each signal with a LOD score greater than 3 was visually inspected and data originating from peak picking errors, thermal noise, elution artifacts, or associated with the void and wash volumes were excluded. Coeluting adducts, fragments, and isotopomers were condensed into their respective parent masses and the intensities for each of the final parent masses were hand-verified to correct for peak picking errors.

### Compound identification

A list of potential compound identities was generated by searching the Madison Metabolomics Consortium Database [27] and the Human Metabolome Database [28] for metabolites matching the QTL-identified masses. Putative IDs were evaluated by ms/ms fragmentation analysis. Final compound identities were confirmed by coelution of the parasite-derived compounds with standards purchased from Sigma and the Proteomics Resource Center at Rockefeller University. The final assignment and quantification steps were conducted by single reaction monitoring (SRM) on a triple quadrupole mass spectrometer.

### Peptidomics data analysis

Peptide assignments were conducted using a comprehensive hemoglobin digestion library loaded into Mascot proteomics software (Matrix Science). Only assignments with mass defects of less than 10 ppm, matching scores greater than nine, and observable peptide signals in all nine of the extracts (N = 3 per genotype) were included. All assignments based on parent masses that mapped to adducts or fragments of hemoglobin peptides were excluded. A custom MAVEN-format standards library was generated using the Mascot results and the extracted ion chromatogram of each assignment was visually inspected and quantified in MAVEN. Peak intensities for each peptide were compiled and aligned to both the hemoglobin α and β primary sequences using custom software written in R.

### Parasite fitness assays

Synchronous cultures of isogenic *pfcrt* allele exchange parasites (C2, C4, C6) were grown to the late trophozoite phase and magnetically purified from uninfected cells using a MACS column. The parasitemia of each enriched sample was determined by microscopy and cell counts were determined by hemocytometer. Mixed culture flasks containing either two (C2, C4; C2, C6) or three (C2, C4, C6) genotypes were constructed at mixing ratios of 50∶50 (C2/C4, C2/C6), 25∶75 (C2/C6), or 50∶15∶35 (C2, C4, C6). Each two-way competition experiment was run as a single biological replicate whereas the three-way competition was replicated in three independent flasks (established from a single seed culture) run in parallel. The entire experimental procedure was repeated a second time using asynchronous populations of parasites. Flasks were maintained continuously for 70 days under standard culturing conditions. Culture flasks were cut back 1∶10 every 48 h (to ∼0.5% parasitemia) and DNA was harvested from the excess parasites via Saponin lysis (0.1%) followed by genomic DNA isolation (DNeasy kit, Qiagen). The *pfcrt* allele present in each sample was PCR amplified (primers: CGAGCGTTATAGAGAATTAG, ACAACATCACCGGCTAAGAA). Products were then Sanger sequenced using independent diagnostic primers (GGCTCACGTTTAGGTGGAGG, ACAACATCACCGGCTAAGAA). Sequencing results were analyzed using online tools from Genewiz and allelic abundances in each flask were quantified using diagnostic single nucleotide polymorphisms in PfCRT amino acid positions 74–76, and 98 (C2: ATG AAT AAA AAC, C4: ATT GAA ACA AGC, C6: ATG AAT ACA GAC, Figure S6).

### Modeling *in vitro* competition

Allele frequencies observed in long-term competition experiments were fit using a custom model of *P. falciparum in vitro* growth. All modeling and regression analyses were conducted using custom software written for the R statistical software environment. Our model makes the following assumptions: 1) long-term changes in allele frequencies follow exponential kinetics, 2) parasite clones can differ with respect to life cycle length, 3) DNA abundance follows a sigmoidal accumulation over the life cycle with peak DNA synthesis occurring mid lifecycle, 4) most of the DNA synthesized in one generation is not amplified in the following generation because not all merozoites successfully reinvade, 5) parasite life cycle synchronicity follows a Gaussian distribution that becomes progressively broader with each generation. Using these assumptions, the relative DNA content expected in mixed culture flasks was modeled for each point in the 70 day competition experiment. Initial differences in allele frequencies were set according to the empirical mixing ratio, then life cycle lengths and exponential growth rates were sampled by grid search. A best-fit multiple regression model was identified by iterative grid searches with progressively finer increments of cycle lengths and growth rates. The custom R code (Text S2 and Text S3) and data table (Table S4) used for this analysis is provided in the supplemental materials.

## Supporting Information

Figure S1 Peptide levels observed in parasites carrying the chloroquine resistant alleles (C6 or C4 derived from HB3 and 7G8 parasite lines, respectively) versus a chloroquine sensitivity allele (derived from HB3) in an isogenic background (GC03, one of the progeny of the HB3×Dd2 cross). The maximum intensity observed for each peptide in the CQS C2 line (generally from the 48 hour point) was used as a reference and all other signals were expressed as a fold change relative to these values. The mean peptide signal is a composite phenotype reporting the average intensity of 12 *pfcrt*-linked signals identified by mQTL.
(PDF)

Figure S2 Concentrations of metabolites observed in Percoll-purified chloroquine sensitive (C2) and chloroquine resistant (C4 and C6) parasites. Concentrations are expressed as the mean µM concentrations present in 1∶4 dilutions of extracts from packed infected cells. Error bars indicate standard deviation of pool replicates (N = 3). All peptides are listed by their standard amino acid abbreviations, 6PGn indicates 6-phosphogluconate. * 6PGn was not linked to PfCRT by QTL and we did not anticipate a significant phenotype for this compound in these lines.
(PDF)

Figure S3 CQR isoforms of PfCRT disrupt hemoglobin α metabolism. Endogenous peptide levels observed in transgenic parasites carrying CQS (C2, Hb3) versus CQR (C4, Dd2; C6, 7G8) alleles of *pfcrt*. Peptides were detected by LC-MS/MS peptidomics and are colored by their mean absolute z score relative to C2 levels.
(PDF)

Figure S4 CQR isoforms of PfCRT disrupt hemoglobin β metabolism. Endogenous peptide levels observed in transgenic parasites carrying CQS (C2, Hb3) versus CQR (C4, Dd2; C6, 7G8) alleles of *pfcrt*. Peptides were detected by LC-MS/MS peptidomics and are colored by their mean absolute z score relative to C2 levels.
(PDF)

Figure S5 Metabolite levels observed in transgenic parasites carrying either CQS (C2) or CQR (C4, C6) *pfcrt* alleles. (**A**) Metabolite intensities for all observed compounds are shown with compounds clustered according to biological function. The 11 peptides listed at the bottom of this figure are the mQTL-identified compounds. Data are plotted as a fold change relative to average signals observed in the C2 lines. Data include six replicates from two independent biological repeats of the experiment. Data are shown as the average response from each biological replicate (1, 2). (**B**) Select central carbon metabolites, amino acids, and glutathione (both oxidized and reduced forms), are shown relative to the tripeptide PEE. These data illustrate the large discrepancy between the peptide phenotype and typical metabolic profiles. Data are plotted as the average ratio relative to signals observed in the C2 lines. Error bars indicate standard deviation.
(PDF)

Figure S6 Sanger sequencing traces showing the abundance of *pfcrt* alleles after 1, 15, and 30 generations in a mixed culture flask containing of CQS (C2) and CQR (C4) parasites.
(PDF)

Figure S7 Actual versus observed abundances of alleles present in artificially mixed samples of DNA. Samples were prepared by mixing DNA isolated from one genotype at various pre-determined ratios with DNA isolated from the other genotypes. Allele frequencies were quantified using Sanger sequencing and plotted as a function of the known mixing ratios.
(PDF)

Figure S8 Eight independent *in vitro* competition experiments between transgenic parasites carrying CQS (C2, Hb3) or CQR (C4, Dd2; C6, 7G8) isoforms of PfCRT. Experiments were conducted with both synchronous (column 1) and asynchronous (column 2) populations of parasites. Mixed culture flasks contained a (**A**) three-way competition between C2, C4 and C6, or (**B, C, D**) two way competitions between each of the lines. Mixed cultures were maintained using standard methods and DNA was harvested every 48 hours.
(PDF)

Figure S9 Parasite growth in rich and amino acid-restricted medium. Cultures were split into normal medium or medium containing isoleucine as the only amino acid, grown for 5 days and quantified by flow cytometry. Fractional growth is expressed as the ratio of the parasitemia in the restricted medium culture to that in the rich medium. Differences between C2 and C6 growth rates were significant by t-test (*p* = 0.0075). Error bars show the standard deviation of n = 3 biological replicates.
(PDF)

Table S1 Peptide signal intensities observed in CQS (C2) or CQR (C4, C6) iRBC extracts. Endogenous peptides were analyzed by nano-flow UPLC MS/MS on a LTQ-Orbitrap and peptides were identified using MASCOT. Each peptide identified by MS/MS fragmentation is listed (column 1) and the signal intensities observed in C2 (columns 2–4), C4 (columns 5–7), and C6 (columns 8–10) are noted.
(CSV)

Table S2 Parasite genotypes used for mQTL analysis. The mQTL analysis reported in this study was computed using custom R statistics code (Text S2 and Text S3) that employees the R/qtl package [55]. This table is formatted to meet the requirements of the R/qtl package. Genotypes (A = Dd2, B = Hb3) are reported at each microsatellite marker. Row one lists the names of each marker, row two lists the chromosome number, row three lists the chromosomal position (in cM), and rows 4–39 list the genotype of Dd2, Hb3, then the 34 progeny of this cross. The names of each progeny line are listed in Table S3 and the order of the rows in this table corresponds to the order of the columns in Table S2. The numeric values in column one of this table are used as a place holder. In this instance, these numbers report the PEE peptide intensities observed in each of the parasite lines.
(CSV)

Table S3 Metabolite phenotypes used for mQTL analysis. The mQTLs reported in this study were computed using custom R statistics code (Text S2 and Text S3) that employees the R/qtl package [55]. This table is formatted to meet the requirements of the R/qtl package. Column one lists the metabolites identified by mQTL. Peptides are listed by their standard abbreviations; Ukn1, Ukn2, and Ukn3 are unknowns; 6PGn stands for six-phosphogluconate. Each subsequent column reports metabolite intensities observed in a parasite line.
(CSV)

Table S4 CQS versus CQR allele frequencies observed in mixed culture flasks. Figure 6 depicts regression curves computed from a custom computer model of population-level allele frequencies expected in *in vitro* competition experiments involving *P. falciparum* (Text S2 and Text S3). This table lists the request input data used to compute the regression statistics listed in Figure 6. Column one shows the each generation (defined as 48 hours), columns 2 and 3 show average allele frequencies plotted in Figure 6A whereas columns 4 and 5 list allele frequencies plotted in Figure 6B.
(CSV)

Text S1 Protocol for preparing metabolomics samples from *P. falciparum* cultures. This file provides a detailed step-by-step protocol for isolating iRBCs and preparing metabolomics samples for LC-MS analysis.
(PDF)

Text S2 Custom R functions for modeling allele frequencies and computing mQTL. This file contains the custom R functions needed to reproduce the analyses described in the text. These functions are called by the code in Text S3.
(PDF)

Text S3 Custom R code for computing allele frequencies and running mQTL analyses. This file uses the custom R functions in Text S2 to model allele frequencies and to run the mQTL analysis.
(PDF)
