# Supplementary figures and images for: Metabolic QTL Analysis Links Chloroquine Resistance in Plasmodium falciparum to Impaired Hemoglobin Catabolism
Source: PLoS Genet. 2014 Jan 2;10(1):e1004085. doi: 10.1371/journal.pgen.1004085 (PMC3879234; doi:10.1371/journal.pgen.1004085)

Fold Change

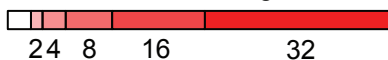

C6

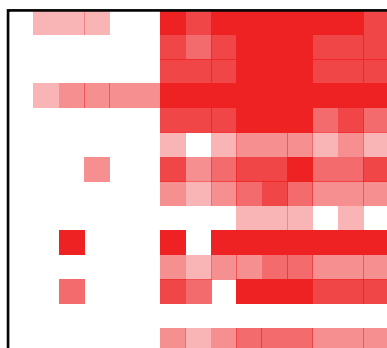

C4

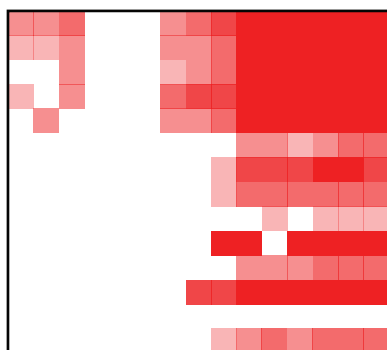

C2

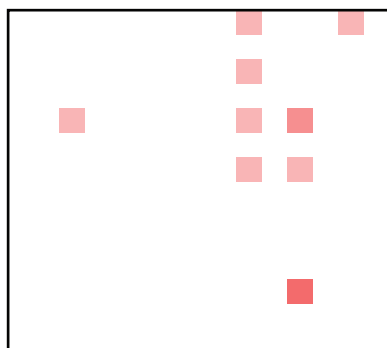

Peptide

0 12 24 36 48

Hours Post Invasion

Supplement: Figure S1 — Peptide levels observed in parasites carrying the chloroquine resistant alleles (C6 or C4 derived from HB3 and 7G8 parasite lines, respectively) versus a chloroquine sensitivity allele (derived from HB3) in an isogenic background (GC03, one of the progeny of the HB3×Dd2 cross). The maximum intensity observed for each peptide in the CQS C2 line (generally from the 48 hour point) was used as a reference and all other signals were expressed as a fold change relative to these values. The mean peptide signal is a composite phenotype reporting the average intensity of 12 pfcrt-linked signals identified by mQTL. (PDF) [file pgen.1004085.s001.pdf]

Concentration ( $\mu$ M) in parasite extracts

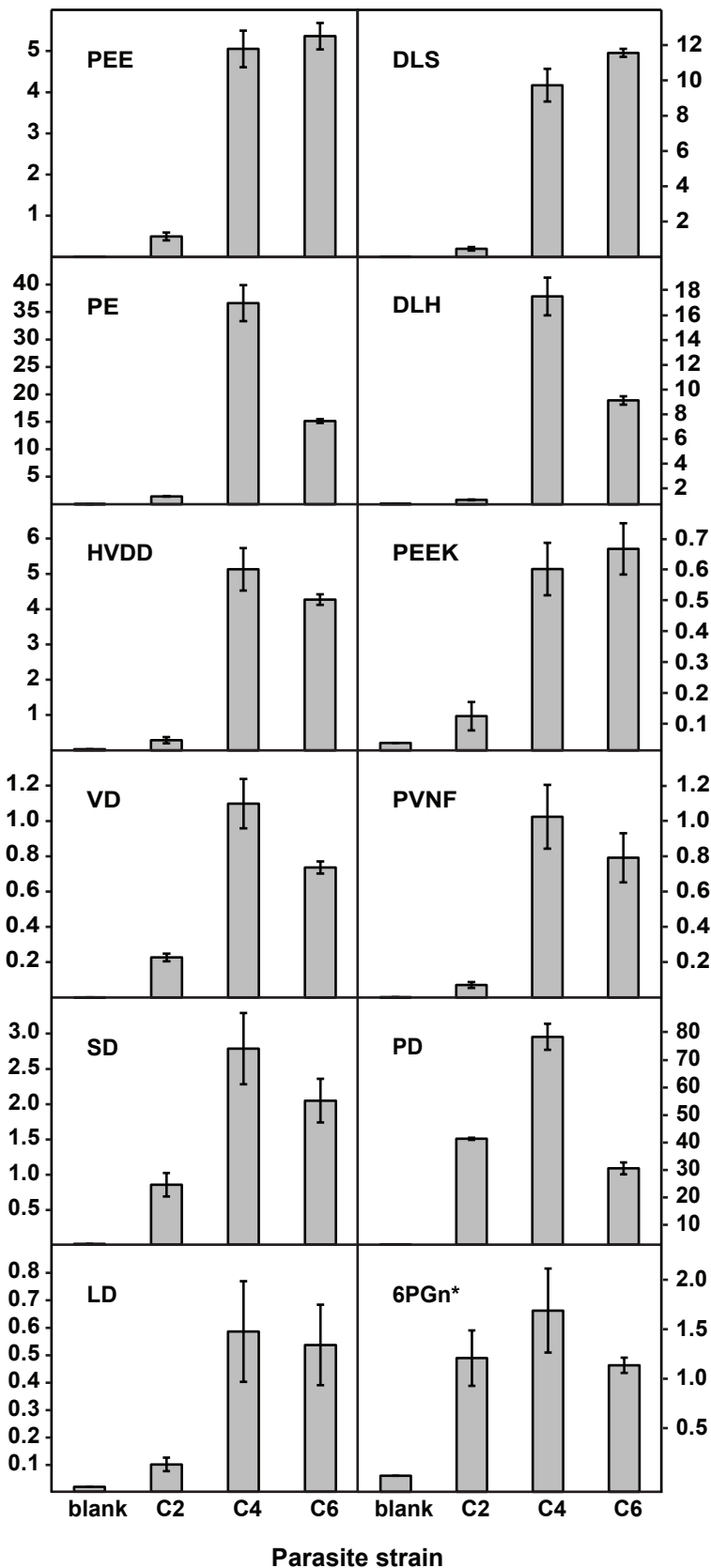

Supplement: Figure S2 — Concentrations of metabolites observed in Percoll-purified chloroquine sensitive (C2) and chloroquine resistant (C4 and C6) parasites. Concentrations are expressed as the mean µM concentrations present in 1∶4 dilutions of extracts from packed infected cells. Error bars indicate standard deviation of pool replicates (N = 3). All peptides are listed by their standard amino acid abbreviations, 6PGn indicates 6-phosphogluconate. * 6PGn was not linked to PfCRT by QTL and we did not anticipate a significant phenotype for this compound in these lines. (PDF) [file pgen.1004085.s002.pdf]

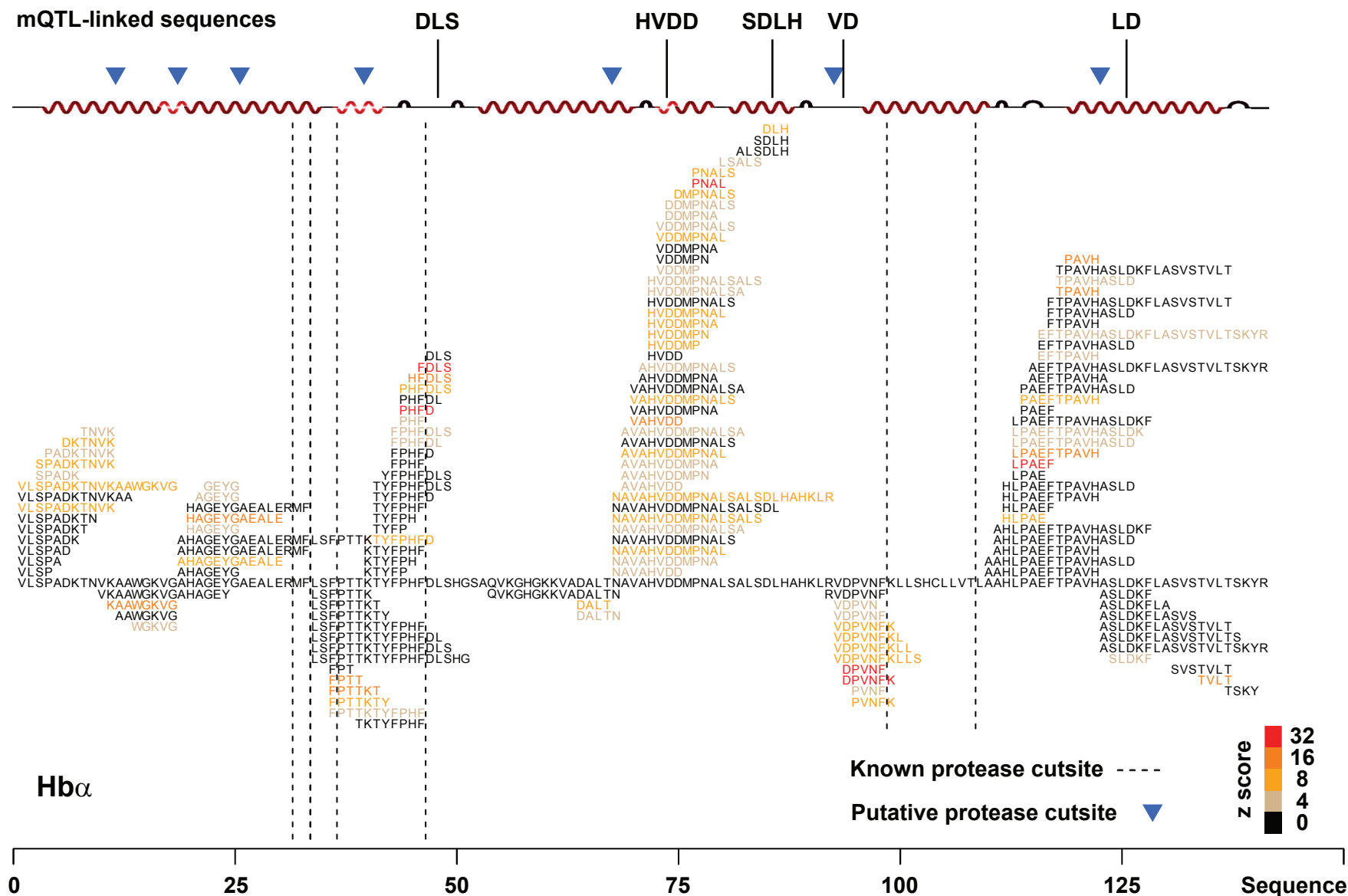

Supplement: Figure S3 — CQR isoforms of PfCRT disrupt hemoglobin α metabolism. Endogenous peptide levels observed in transgenic parasites carrying CQS (C2, Hb3) versus CQR (C4, Dd2; C6, 7G8) alleles of pfcrt. Peptides were detected by LC-MS/MS peptidomics and are colored by their mean absolute z score relative to C2 levels. (PDF) [file pgen.1004085.s003.pdf]

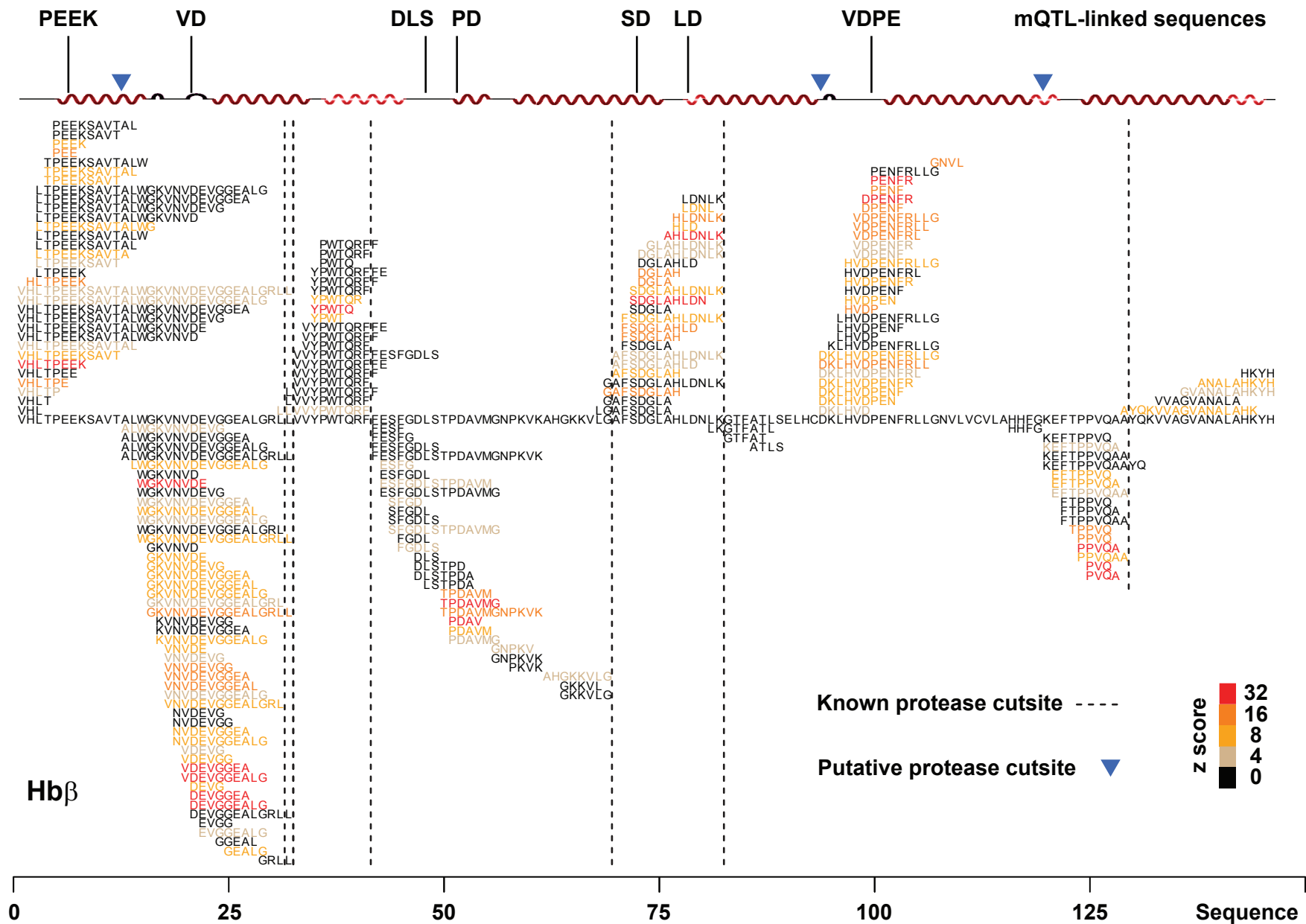

Supplement: Figure S4 — CQR isoforms of PfCRT disrupt hemoglobin β metabolism. Endogenous peptide levels observed in transgenic parasites carrying CQS (C2, Hb3) versus CQR (C4, Dd2; C6, 7G8) alleles of pfcrt. Peptides were detected by LC-MS/MS peptidomics and are colored by their mean absolute z score relative to C2 levels. (PDF) [file pgen.1004085.s004.pdf]

A

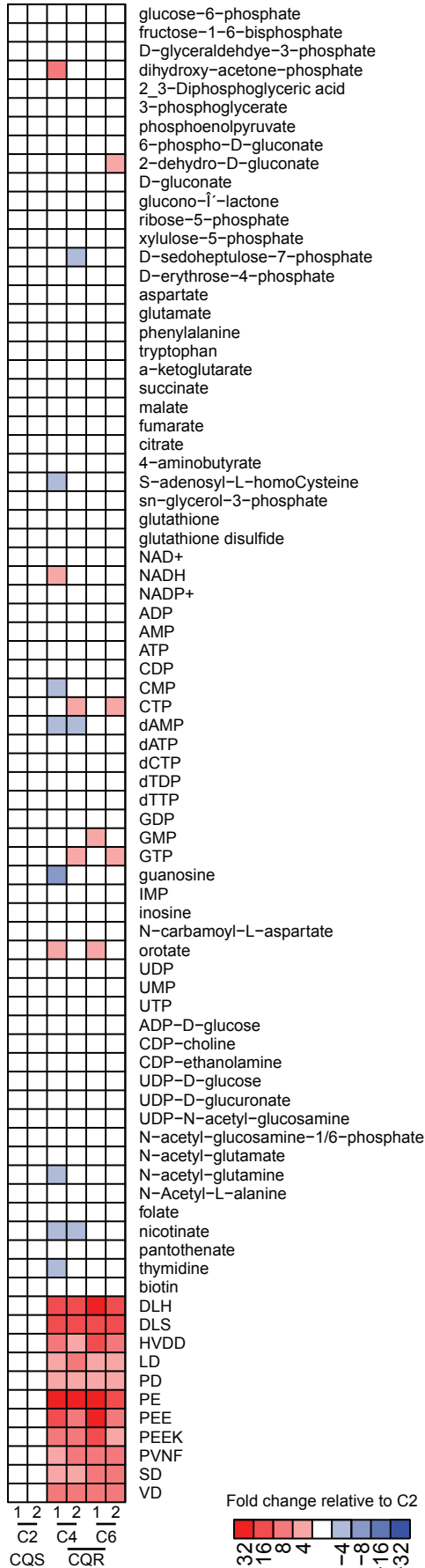

B

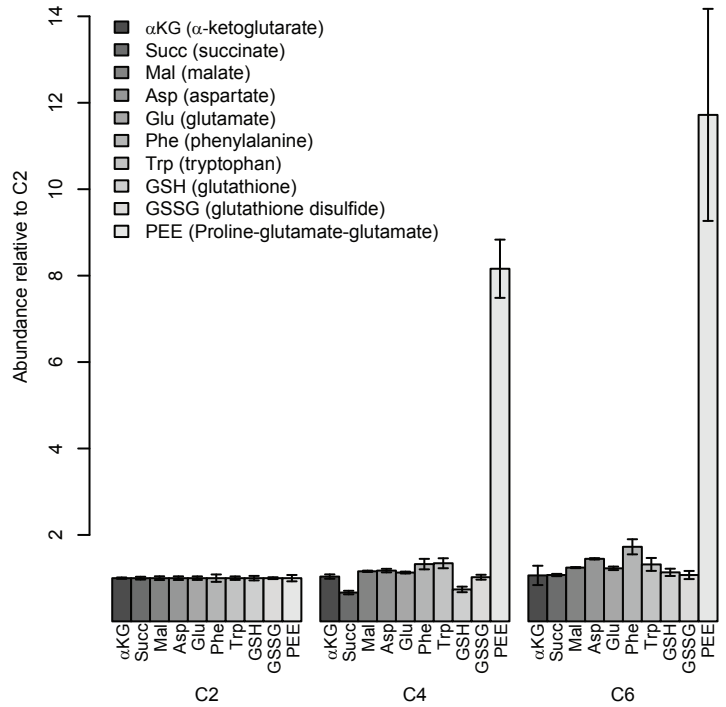

Supplement: Figure S5 — Metabolite levels observed in transgenic parasites carrying either CQS (C2) or CQR (C4, C6) pfcrt alleles. (A) Metabolite intensities for all observed compounds are shown with compounds clustered according to biological function. The 11 peptides listed at the bottom of this figure are the mQTL-identified compounds. Data are plotted as a fold change relative to average signals observed in the C2 lines. Data include six replicates from two independent biological repeats of the experiment. Data are shown as the average response from each biological replicate (1, 2). (B) Select central carbon metabolites, amino acids, and glutathione (both oxidized and reduced forms), are shown relative to the tripeptide PEE. These data illustrate the large discrepancy between the peptide phenotype and typical metabolic profiles. Data are plotted as the average ratio relative to signals observed in the C2 lines. Error bars indicate standard deviation. (PDF) [file pgen.1004085.s005.pdf]

|    |    |   |   |    |   |   |    |   |   |
|----|----|---|---|----|---|---|----|---|---|
|    | 74 |   |   | 75 |   |   | 76 |   |   |
| C2 | A  | T | G | A  | A | T | A  | A | A |
| C4 |    |   | T | G  |   | A |    | C |   |

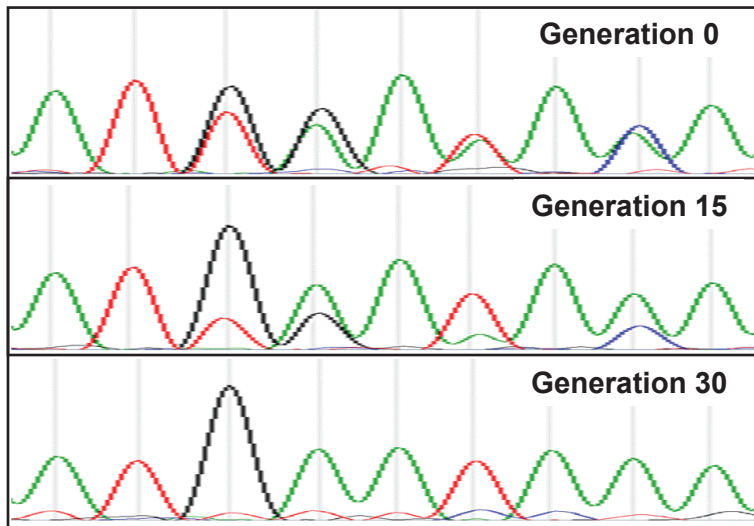

— A  
— T  
— G  
— C

*pfcrt* DNA sequence in mixed C2/C4 cultures

Supplement: Figure S6 — Sanger sequencing traces showing the abundance of pfcrt alleles after 1, 15, and 30 generations in a mixed culture flask containing of CQS (C2) and CQR (C4) parasites. (PDF) [file pgen.1004085.s006.pdf]

Abundance observed by quantitative DNA sequencing

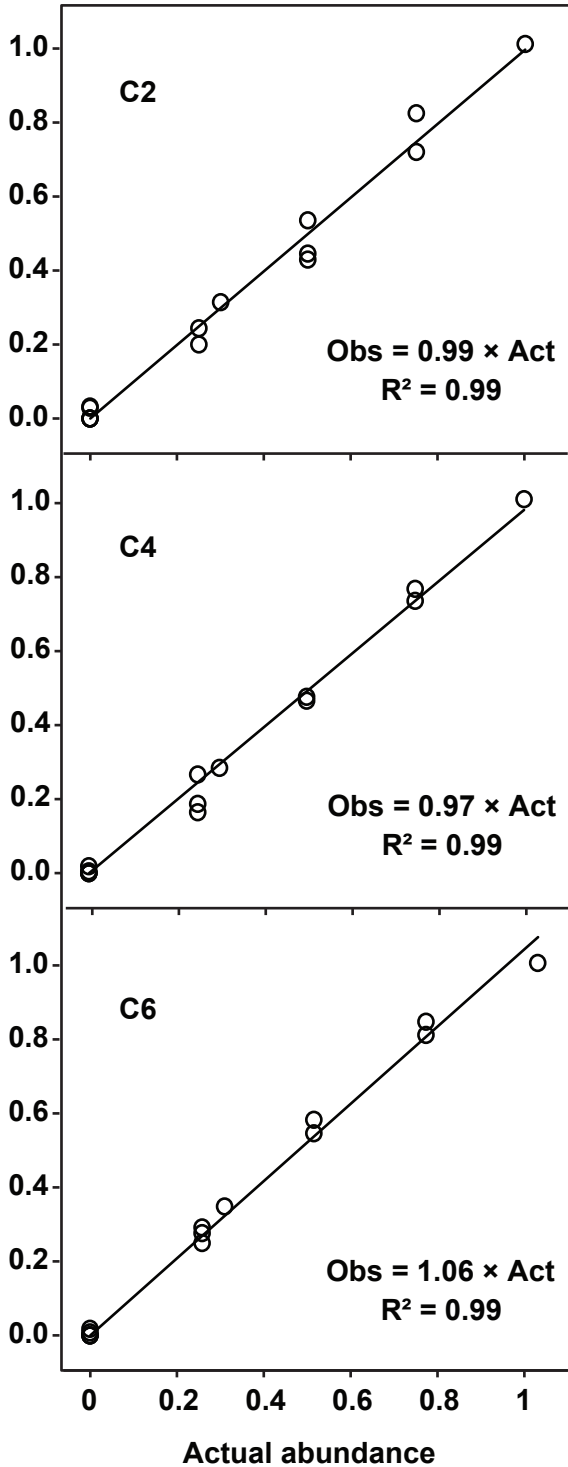

Supplement: Figure S7 — Actual versus observed abundances of alleles present in artificially mixed samples of DNA. Samples were prepared by mixing DNA isolated from one genotype at various pre-determined ratios with DNA isolated from the other genotypes. Allele frequencies were quantified using Sanger sequencing and plotted as a function of the known mixing ratios. (PDF) [file pgen.1004085.s007.pdf]

## Synchronous Cultures

## Asynchronous Cultures

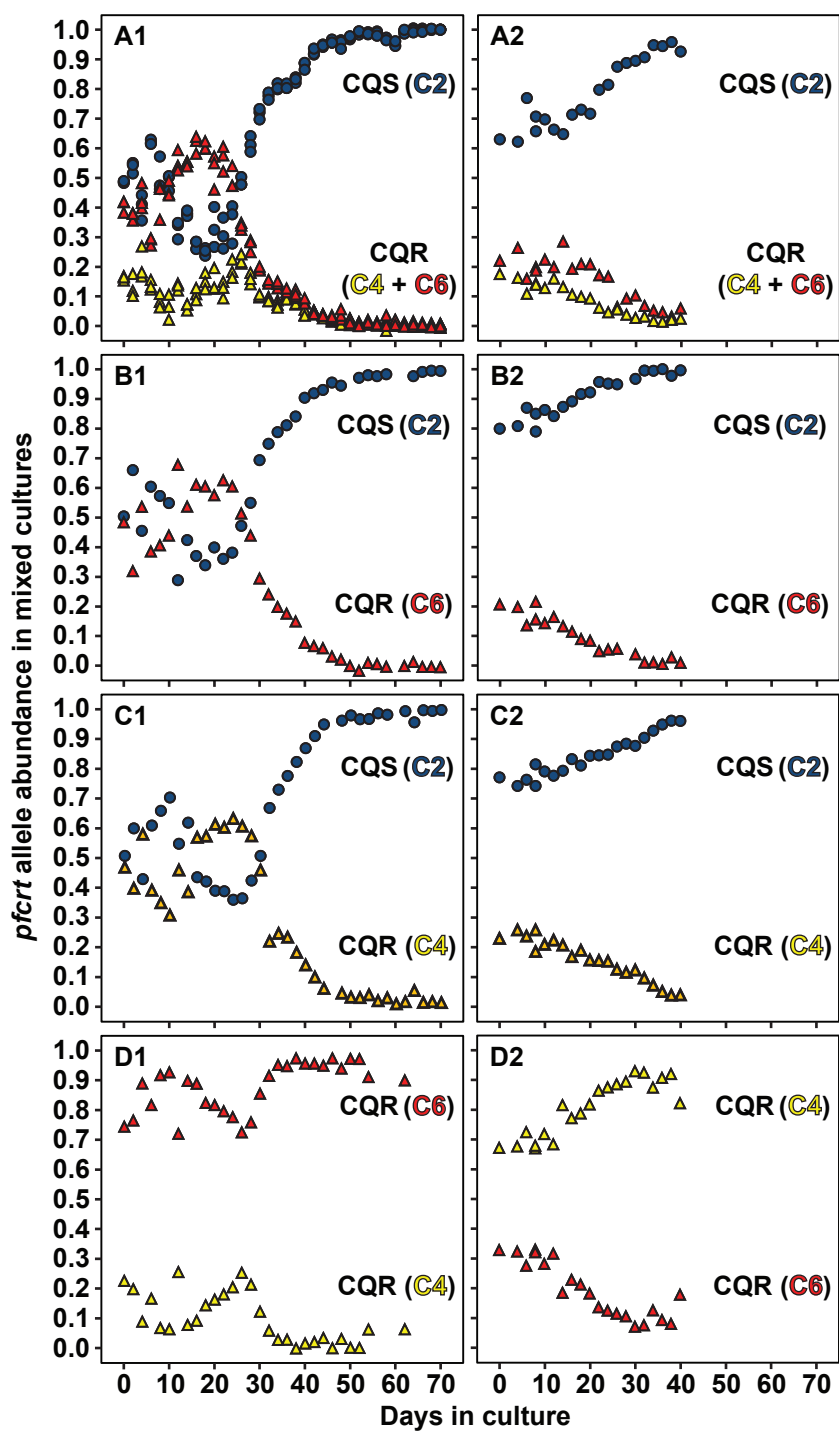

Supplement: Figure S8 — Eight independent in vitro competition experiments between transgenic parasites carrying CQS (C2, Hb3) or CQR (C4, Dd2; C6, 7G8) isoforms of PfCRT. Experiments were conducted with both synchronous (column 1) and asynchronous (column 2) populations of parasites. Mixed culture flasks contained a (A) three-way competition between C2, C4 and C6, or (B, C, D) two way competitions between each of the lines. Mixed cultures were maintained using standard methods and DNA was harvested every 48 hours. (PDF) [file pgen.1004085.s008.pdf]

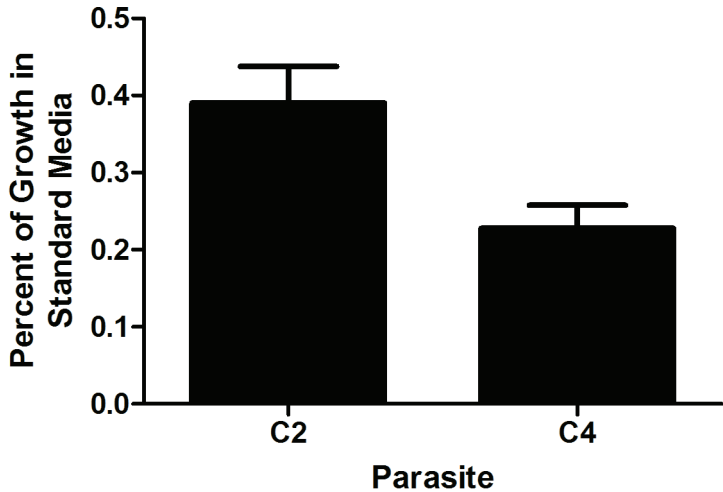

Supplement: Figure S9 — Parasite growth in rich and amino acid-restricted medium. Cultures were split into normal medium or medium containing isoleucine as the only amino acid, grown for 5 days and quantified by flow cytometry. Fractional growth is expressed as the ratio of the parasitemia in the restricted medium culture to that in the rich medium. Differences between C2 and C6 growth rates were significant by t-test (p = 0.0075). Error bars show the standard deviation of n = 3 biological replicates. (PDF) [file pgen.1004085.s009.pdf]
