# Supplementary material for: Metabolic QTL Analysis Links Chloroquine Resistance in Plasmodium falciparum to Impaired Hemoglobin Catabolism
Source: PLoS Genet. 2014 Jan 2;10(1):e1004085. doi: 10.1371/journal.pgen.1004085 (PMC3879234; doi:10.1371/journal.pgen.1004085)
Supplement: Text S1 — Protocol for preparing metabolomics samples from P. falciparum cultures. This file provides a detailed step-by-step protocol for isolating iRBCs and preparing metabolomics samples for LC-MS analysis. (PDF) [file pgen.1004085.s014.pdf]

Recommended Sample Preparation Protocol for MS Metabolomics

One synchronous 50 mL flask at 2% hematocrit and 10% parasitemia yields ~ 3 MS samples

Materials Need Prior to Sample Preparation

1) Synchronized cultures of 36-42 h trophozoites (<8 h synchronicity window) at 10% parasitemia
2) One bucket of ice/water slurry
3) RPMI
4) iRBC isolation materials (either A or B)
   A. 70% and 30% Isotonic Percoll made from 10X RPMI
      Prepare Percoll gradient tubes for each genotype. For large samples (> 3 ml packed cell volume), carefully layer 10 mL 30% Percoll on top of 15 mL of 70% Percoll in a 50 mL Falcon tube. For smaller sample, use 4 mL of 30% and 5 mL of 70% Percoll in a 15 mL Falcon tube.
   B. Wash magnetic column (Macs CS) in 10 volumes PBS, then fill with RPMI, and place in magnet.
5) 90% methanol / ddH_2O (HPLC grade reagents)
   Prepare 1 mL aliquotes for media samples in 1.5 ml Eppendorf tubes and store on ice.
6) Microfuge tubes

Preparing iRBC-enriched samples by Percoll

1) Spin cultures down at 3,500 x g for 5 min at room temperature
2) Aspirate off medium and resuspend pellet at 20% Hct in room temperature RPMI
   Carefully layer 30% Hct blood over the 30% / 70% Percoll gradient
3) Spin cultures down at 3,500 x g for 15 min at room temperature
4) Troph/schizonts form a band at the 30% / 70% interface. Carefully aspirate off the RPMI and 30% Percoll layers. Collect the schizont band with a P1000 pipette and wash the cells in 50 mL RPMI.

Preparing iRBC-enriched samples by magnet

1) Spin cultures down at 3,500 x g for 5 min at room temperature
2) Aspirate off medium and resuspend pellet at 7% Hct in room temperature RPMI
3) Flow culture over magnetic column and wash RPMI until flow through is clear (~ 4X column volume), then wash with one additional column volume.
4) Remove column from magnet and elute iRBCs into a 15 ml falcon tube

Experimental setup

1) Spin cultures down at 3,500 x g for 5 min at room temperature
2) Resuspend at exactly 0.4% Hct (20 µL iRBC / 5 ml media). Use the pipette dial back method to measure the volume of a 50% Hct iRBC 50% suspension, then adjust to 0.4% Hct. Isotopic labels can be added at this point.
3) Aliquot 5 ml samples into 6-well culture plates.
4) Harvest 20 µL of each iRBC cell suspension and dilute to 200 µL for cell counting on a hemocytometer.
5) Incubate parasitized cells for 2 h at 37 °C

Metabolite extraction- should be performed in a 4 °C cold room

1) Transfer the 6 well plates of samples directly from incubator to an ice/water slurry
2) Dislodge cells from the bottom of wells by pipetting up and down (DO NOT bubble sample) and transfer samples to 15 mL centrifuge tubes
3) Spin samples down at 3,500 x g for 5 min at 4 °C
4) (Optional) Harvest 50 µL of medium and combine each with 1 mL of 90% methanol
5) Aspirate off the remaining medium and discard; do not disturb the cell pellet
6) Pipette 1 mL of 90% methanol over cell pellet and immediately vortex for 2 seconds
7) Centrifuge (10 min at 10,000 × g) and transfer supernatants to fresh microfuge tubes.
8) Store at -80 °C until MS analysis
   Blow off methanol under N_2 and resuspend extracts at a 1:6 dilution in HPLC-grade ddH_2O. Adjust the resuspension volume with the hemocytometer-based cell counts to achieve samples with a consistent dilution of 0.85 x 10^6 cells per µL ddH_2O.
