# Supplementary material for: Metabolic QTL Analysis Links Chloroquine Resistance in Plasmodium falciparum to Impaired Hemoglobin Catabolism
Source: PLoS Genet. 2014 Jan 2;10(1):e1004085. doi: 10.1371/journal.pgen.1004085 (PMC3879234; doi:10.1371/journal.pgen.1004085)
Supplement: Text S2 — Custom R functions for modeling allele frequencies and computing mQTL. This file contains the custom R functions needed to reproduce the analyses described in the text. These functions are called by the code in Text S3. (PDF) [file pgen.1004085.s015.pdf]

```
###############################################################################
##
###############################################################################
##
##
##
##
##
##    rPlasmo tools, Tools for viewing and analyzing malaria data.
##
##    Copyright (C) 2013 Ian A. Lewis under GPL-3
##
##
##
##    This program is free software: you can redistribute it and/or modify
##
##    it under the terms of the GNU General Public License as published by
##
##    the Free Software Foundation, either version 3 of the License, or
##
##    any later version.
##
##
##
##    This program is distributed in the hope that it will be useful,
##
##    but WITHOUT ANY WARRANTY; without even the implied warranty of
##
##    MERCHANTABILITY or FITNESS FOR A PARTICULAR PURPOSE.  See the
##
##    GNU General Public License for more details.
##
##
##
##    A copy of the GNU General Public License can be found at:
##
##    www.r-project.org/Licenses/GPL-3
##
##
##
##
##
###############################################################################
##
###############################################################################
##
```

```
################################################################################
##
################################################################################
##
##               Functions for modeling parasite growth
##
################################################################################
##
################################################################################
##

## User function to read in CSV files using a file choose window
readPeak <- function( inPath = file.choose()){
        return(read.csv( inPath, head = TRUE, stringsAsFactors = FALSE))
}


## User function for fitting observed competition data
## obs ñ numeric vector, describes allele abundance over the experiment
## pop1 ñ numeric, the starting allele frequency of the population
## lc1 ñ numeric, the life cycle length for the observed population
## lc2 ñ numeric, the life cycle length for the competing population
## r1 ñ numeric, differential growth rate of the populations
## t0 ñ numeric, life cycle state in hours at the start of the experiment
## sc ñ numeric, the X-fold amplification of the genome per generation
## pSync ñ numeric, Gaussian half height parasite synchronicity in hours
## N   - integer, number of fitting iterations
## rSample ñ logical, FALSE samples the complete input grid, TRUE randomly
##              samples N points
## syncC ñ logical, FALSE sets parasite synchronicity to 0
## returns a table with fitting parameters and RSQ
fitComp <- function(obs, pop1, lc1, lc2, r1, t0, sc, pSync,
                N = 10000, rSample = T, syncC = F ){

        if( missing(obs) )
                stop( 'A vector of observed populations must be provided')

        ## Set sampling parameters
        if( missing(pop1) )
                pop1 <- ((1:19) * 5) / 100
        pop2 <- 1 - pop1
        if( missing(lc1) )
                lc1 <- 38:58 #lc1 <- 45:51
        if( missing(lc2) )
                lc2 <- 38:58 #lc2 <- 45:51
        if( missing(r1) )
```

```
		r1 <- (-5:5) *2 #r1 <- .050+(0:10)/1000
	if( missing(t0) )
		t0 <- 38:48  #t0 <- 41:43
	if( missing(sc) )
		sc <- 1:10 #sc <- (-4:4)/4 + 4
	if( missing(pSync) )
		pSync <- 1:10 #pSync <- (1:4)*2
	if( !syncC )
		pSync <- 0

	fitT <- unique(expand.grid( pop1 = pop1, pop2 = pop2, lc1 = lc1,
					lc2=lc2, r1 = r1, t0=t0, sc=sc,
pSync=pSync ))
	if( rSample && nrow(fitT) > (N*2) )
		fitT <- fitT[ sort(sample(1:nrow(fitT), N)), ]

	if( !syncC )
		print( paste('Processing time:', round((nrow(fitT)/36)/60,2),
'minutes' ))
	else
		print( paste('Processing time:', round((nrow(fitT)*18)/60,2),
'minutes' ))
	flush.console()

	## Calculate regression curves
	print('Calculating regression curves ...')
	flush.console()
	fitData <- list()
	for( i in 1:nrow(fitT) ){
		fitData[[i]] <- compGrowth(
				pop1 = fitT$pop1[i],
				pop2 = fitT$pop2[i],
				lc1 = fitT$lc1[i],
				lc2 = fitT$lc2[i],
				r1 = fitT$r1[i],
				t0 = fitT$t0[i],
				sc = fitT$sc[i],
				pSync = fitT$pSync[i],
				g = length(obs) - 1, syncC = syncC )
	}

	## Measure the fit
	print('Calculating RSQ ...')
	flush.console()
	RSQ1 <- list()
	if( syncC ){
		for( i in 1:length(fitData) )
```

```
                        RSQ1[[i]] <- RSQ( obs = obs, fit = fitData[[i]]
$DNAS1 )
        }else{
                for( i in 1:length(fitData) )
                        RSQ1[[i]] <- RSQ( obs = obs, fit = fitData[[i]]$DNA1 )
        }
        fitT$RSQ <- unlist(RSQ1)

        return( fitT )
}

## Internal function for modeling for competitive growth
## pop1 - numeric - fraction of population 1 at time 0
## pop2 - numeric - fraction of population 2 at time 0
## lc1  - numeric - life cycle length pop1 (hours)
## lc2  - numeric - life cycle length pop2 (hours)
## r1    - numeric - differential growth rate
## g    - numeric - number of generations (assumes 48h/g)
## t0 ñ numeric, life cycle state at the start of the experiment (e.g. 42 h)
## sc ñ numeric, the X-fold amplification of the genome per generation
## ri ñ numeric, reinvasion efficiency
## ri ñ numeric, reinvasion efficiency
## pSync ñ numeric, Gaussian half height parasite synchronicity in hours
## syncC ñ logical, FALSE sets parasite synchronicity to 0
compGrowth <- function(pop1 = .5, pop2 = .5, lc1 = 47, lc2 = 49, r1 = .065,
                r2 = -r1, g = 35, t0 = 43, sc = 8, ri = 1/sc, pSync = 1,
sample48h = T,
                syncC = T){

        ## Define experimental setup
        t0 = t0 - 1
        Tte <- (-(48*g):(48*g*2)) - 1
        Ge <- Tte %/% 48
        Te <- Tte %% 48

        ## Calculate number of generations for each population
        g1 =(Tte+t0) %/% lc1
        g2 =(Tte+t0) %/% lc2

        ## Calculate population sizes
        out <- data.frame(
                        Tte = Tte,
                        Ge = Ge,
                        Te = Te,
                        g1 = g1,
                        t1 =((Tte+t0) %% lc1) + 1,
                        p1 =compG(p1 = pop1, g = g1, r1 = 1+r1 )$p1,
```

```
                    DNA1 = rep(NA, length(g1)),
                    g2 = g2,
                    t2 =((Tte+t0) %% lc2) + 1,
                    p2 = compG(p2 = pop2, g = g2, r2 = 1+r2 )$p2,
                    DNA2 = rep(NA, length(g1))
        )

        ## Calculate amount of DNA for each population
        D1 <- DNAr(t = 1:lc1, lc = lc1, sc = sc, ri = ri)
        D2 <- DNAr(t = 1:lc2, lc = lc2, sc = sc, ri = ri)
        out$DNA1 <- D1[match(out$t1, 1:lc1)]
        out$DNA2 <- D2[match(out$t2, 1:lc2)]
        out$DNA1 <- (out$DNA1 * out$p1)
        out$DNA2 <- (out$DNA2 * out$p2)

        ## Adjust for synchronicity
        if( syncC ){
                idx1 <- which( out$Tte == 0)
                idx2 <- rev(which( out$Ge <= g ))[1]
                out$DNAS1 <- out$DNAS2 <- NA
                syncT <- sync(c = 2)$p
                N <- (length(syncT) - 1)/2
                for( i in  idx1:idx2 ){
                        syncT <- sync( c = out$Ge[i]/g * 6 + pSync )$p
                        out[i,]$DNAS1 <- sum(out$DNA1[ (i - N) : (i + N)] *
syncT)
                        out[i,]$DNAS2 <- sum(out$DNA2[ (i - N) : (i + N)] *
syncT)
                }

                ## Calculate fractional pool
                TOT <- out$DNAS1  + out$DNAS2
                out$DNAS1 <- out$DNAS1 / TOT
                out$DNAS2 <- out$DNAS2 / TOT
        }

        ## Calculate the fractional pool
        TOT <- out$DNA1 + out$DNA2
        out$DNA1 <- out$DNA1 / TOT
        out$DNA2 <- out$DNA2 / TOT

        out <- out[ out$Tte >= 0 & out$Ge <= g,]
        if( !sample48h )
                return(out)
        return(out[ out$Te == 0,])
}
```

```
## Internal model for exponential growth
compG <- function( p1 = 0.5, p2 = 1 - p1, r = .08, r1 = 1 + r, r2 = 1 - r,
                g = 1:35){

        P1T <- p1 * exp( r1 * g)
        P2T <- p2 * exp( r2 * g)
        tot <- P1T + P2T

        return( data.frame(g = g, p1 = P1T, p2 = P2T, tot ))
}

## Internal model for DNA replication
DNAr <- function(lc = 48, t = 1:lc, sc = 4, ri = .25){
        return( 1/(1+exp(-t/4 + lc/8)) * (sc - ri) + ri )
}

## Internal model for population synchronicity
sync <- function(x = -240:240, t = 0, c = 4 ){
        gc <- exp(-(x-t)^2/(2*c^2))
        tot <- sum(exp(-(x-t)^2/(2*c^2)))
        return( data.frame(t = x, p = gc/tot) )
}

## Internal function to calculate RSQ for each fit
RSQ <- function( obs, fit){

        if( any(is.na(fit)) )
                return(NA)
        mod <- summary( lm(fit ~ obs) )
        return(mod$r.squared * (abs(mod$coefficients[2,1]) / mod
$coefficients[2,1]) )
}



##############################################################################
##
##############################################################################
##
##                        mQTL related functions
##
##############################################################################
##
##############################################################################
##
```

```
## Read markerlist
readMark <- function(){
        require(qtl)
        markerPath <- file.choose()
        markerList <- read.cross("csv", dirname(markerPath),
basename(markerPath),
                        genotypes = c("A", "B"))
}


## Function for mQTL analysis
mzQTL <- function( pheno, markerList, plotQTL = FALSE, norm = TRUE,
                nPerm = 1000, covar, summary = F, c = 100 ){

        require(qtl)

        ## Variance stabilize data
        phenoNames <- pheno[,1]
        mzTab <- pheno[,-1]
        if( norm )
                mzTab <- log(mzTab + c )

        ## Make sure covar list is the right length
        if( !is.null(covar) && length(covar) !=
length(unlist(markerList[[2]])) )
                stop( 'The covarance list is the wrong length')

        ## Prep data for QTL analysis
        markers <- calc.genoprob(markerList, step=1, error.prob=0.001)
        markers <- sim.geno(markers, step=1, n.draws=64, error.prob=0.001)

        ## Run QTL analysis
        lodList <- list()
        alpha05 <- list()

        for ( i in 1:nrow(mzTab) ){
                markers[[2]] <- t(mzTab[i,])
                try( out <- scanone(markers, method = 'hk', addcovar = covar),
silent = TRUE)
                suppressWarnings(
                                if(class(out) == "try-error"){
                                        lodList[[i]] <- NA
                                                next
                                } )

                lodList[[i]] <- out
```

```
		if( summary == T)
			next

		# Find significance by permutation
		try( operm <- scanone(markers, method = 'hk', n.perm=nPerm,
addcovar = covar),
					silent = TRUE)
		suppressWarnings(if(class(operm) == "try-error"){
					alpha05[[i]] <- NA
					next
				} )
		alpha05[[i]] <- operm
	}

	names(lodList) <- phenoNames

	if( summary ){
		chr <- sapply(lodList, function(x) max(x)[[1]]  )
		pos <- sapply(lodList, function(x) max(x)[[2]]  )
		lod <- sapply(lodList, function(x) max(x)[[3]]  )
		return( data.frame(chr, pos, lod, stringsAsFactors = F))

	}

	names(alpha05) <- phenoNames
	return(list(lodList, alpha05))
}
```
