# Supplementary material for: Metabolic QTL Analysis Links Chloroquine Resistance in Plasmodium falciparum to Impaired Hemoglobin Catabolism
Source: PLoS Genet. 2014 Jan 2;10(1):e1004085. doi: 10.1371/journal.pgen.1004085 (PMC3879234; doi:10.1371/journal.pgen.1004085)
Supplement: Text S3 — Custom R code for computing allele frequencies and running mQTL analyses. This file uses the custom R functions in Text S2 to model allele frequencies and to run the mQTL analysis. (PDF) [file pgen.1004085.s016.pdf]

```
##############################################################################
##
##############################################################################
##
##
##
##
##
##    rPlasmo tools, Tools for viewing and analyzing malaria data.
##
##    Copyright (C) 2013 Ian A. Lewis under GPL-3
##
##
##
##    This program is free software: you can redistribute it and/or modify
##
##    it under the terms of the GNU General Public License as published by
##
##    the Free Software Foundation, either version 3 of the License, or
##
##    any later version.
##
##
##
##    This program is distributed in the hope that it will be useful,
##
##    but WITHOUT ANY WARRANTY; without even the implied warranty of
##
##    MERCHANTABILITY or FITNESS FOR A PARTICULAR PURPOSE.  See the
##
##    GNU General Public License for more details.
##
##
##
##    A copy of the GNU General Public License can be found at:
##
##    www.r-project.org/Licenses/GPL-3
##
##
##
##
##
##############################################################################
##
##############################################################################
##
```

```
################################################################################
##
##                     Code for computing mQTL
##
################################################################################
##

## Read input files
markerList <- readMark() ## QTL_genotype.csv file
pheno <- readPeak() ## QTL_phenotype.csv file
covar <- c(1, 2, 5, 6, 5, 1, 6, 1, 6, 2, 2, 4, 2, 5, 3, 4, 1, 5, 1,
           6, 2, 2, 4, 3, 1, 5, 3, 5, 3, 1, 4, 3, 1, 3, 4, 3)

## Compute QTLs
final <- mzQTL(pheno = pheno, markerList = markerList, covar = covar)
mzAlpha <- c( 0.05, 0.01, 0.001, 0.05 / (nrow(pheno) - 2) )
alphaT <- matrix( NA, nrow = nrow(pheno), ncol = length(mzAlpha) )
for( i in 1:length(mzAlpha) ){
  alphaT[,i] <- sapply(final[[2]], function(x) summary(x, alpha =
mzAlpha[i]) )
}
alphaT <- data.frame(alphaT)
names(alphaT) <- mzAlpha
row.names(alphaT) <- pheno$compound
chr <- as.numeric(as.vector(unlist(sapply(final[[1]],
        function(x) max(x)[1]))))
pos <- as.numeric(as.vector(unlist(sapply(final[[1]],
        function(x) max(x)[2]))))
lod <- as.numeric(as.vector(unlist(sapply(final[[1]],
        function(x) max(x)[3]))))
pVal <- rep(NA, length(lod))
for( i in 1:length(final[[1]]))
  pVal[i] <- min(summary(final[[1]][[i]], perms = final[[2]][[i]], pvalues =
T)$pval)


## Generate summary
cpdSummary <- data.frame(
        compound = names(final[[1]]),
        chr, pos, lod, pVal, stringsAsFactors  = F)
cpdSummary <- cbind(cpdSummary, alphaT)


################################################################################
##
##                   Code for modeling parasite growth
```

```
##
################################################################################
##

## Read in observed competition data and fitting parameters
dat <- readPeak() ## population_summary.csv file

## Generate 50,000 random sampling points for grid search
fitR  <- fitComp( obs = dat$S_1, pop1 = .5, syncC = F, N = 50000)

## Final fit used for Figure 6A (50:50 population)
fitF1 <- fitComp(obs = dat$S_1,
                pop1 = .5,
                lc1 = 47,
                lc2 = 49,
                t0 = 43,
                r1 = .065,
                sc = 8,
                pSync = 1,
                rSample = F,
                syncC = T
)

## Final fit used for Figure 6B (25:75 population)
fitF2 <- fitComp(obs = dat$S_2,
                pop1 = .25,
                lc1 = 47,
                lc2 = 49,
                t0 = 43,
                r1 = .065,
                sc = 8,
                pSync = 1,
                rSample = F,
                syncC = T
)
```
